# Metabolic Disorders in the Transition Period Indicate that the Dairy Cows’ Ability to Adapt is Overstressed

**DOI:** 10.3390/ani5040395

**Published:** 2015-10-09

**Authors:** Albert Sundrum

**Affiliations:** Department of Animal Nutrition and Animal Health, University of Kassel, Nordbahnhofstraße 1a, D-37213 Witzenhausen, Germany; E-Mail: Sundrum@uni-kassel.de; Tel.: +49-561-804-1710; Fax: +49-561-804-1581

**Keywords:** animal science, allostasis, autopoiesis, dairy cows, downward causation, farm management, living conditions, metabolic load, production diseases

## Abstract

**Simple Summary:**

Metabolic disorders are a key problem in the transition period of dairy cows and often appear before the onset of further health problems. Problems derive from difficulties animals have to adapt to large variations and disturbances occurring both outside and inside the organism. A lack of success in solving these issues may be due to predominant approaches in farm management and agricultural science, dealing with such disorders as merely negative side effects. Instead, a successful adaptation of animals to their living conditions should be seen as an important end in itself. Both farm management and agricultural sciences should support animals in their ability to cope with nutritional and metabolic challenges by employing a functional and result-driven approach.

**Abstract:**

Metabolic disorders are a key problem in the transition period of dairy cows and often appear before the onset of further health problems. They mainly derive from difficulties the animals have in adapting to changes and disturbances occurring both outside and inside the organisms and due to varying gaps between nutrient supply and demand. Adaptation is a functional and target-oriented process involving the whole organism and thus cannot be narrowed down to single factors. Most problems which challenge the organisms can be solved in a number of different ways. To understand the mechanisms of adaptation, the interconnectedness of variables and the nutrient flow within a metabolic network need to be considered. Metabolic disorders indicate an overstressed ability to balance input, partitioning and output variables. Dairy cows will more easily succeed in adapting and in avoiding dysfunctional processes in the transition period when the gap between nutrient and energy demands and their supply is restricted. Dairy farms vary widely in relation to the living conditions of the animals. The complexity of nutritional and metabolic processes and their large variations on various scales contradict any attempts to predict the outcome of animals’ adaptation in a farm specific situation. Any attempts to reduce the prevalence of metabolic disorders and associated production diseases should rely on continuous and comprehensive monitoring with appropriate indicators on the farm level. Furthermore, low levels of disorders and diseases should be seen as a further significant goal which carries weight in addition to productivity goals. In the long run, low disease levels can only be expected when farmers realize that they can gain a competitive advantage over competitors with higher levels of disease.

## 1. Introduction

Dairy cows with high genetic merits live under quite heterogeneous nutritional and environmental conditions. Nevertheless, they are capable of adapting and maintaining their internal organization in the face of changes and disturbances, while simultaneously producing high milk yields. However, individual animals differ tremendously in their adaptive success. Adaptation to changes and disturbances in the environment requires regulations on different scales within the organism and follows a common purpose: to survive as long as possible [1,2]. Thus, adaptation is a functional and target-oriented process which can be assessed in the long run by measuring longevity. In the short run, signs of metabolic and fertility disorders as well as subclinical and clinical diseases indicate varying degrees of adaptability. When disorders occur, it is an illustration that animals have difficulties in coping with external and internal conditions, endangering their own capacity to survive [3]. As farm animals cannot escape their living conditions, their ability to cope depends on the extent their environment has an impact on them and on their level of responsiveness in order to maintain or re-establish a homeostatic state. Any stimulus (external or internal) that challenges homeostasis can be viewed as a stressor [4]. Changes in biological function occur as the animal attempts to respond to stressors and associated challenges.

Over the past decades, the focus of the dairy industry has been on maximizing milk yield, thereby creating a “nutrient highway” for nutrients from the daily feed and body reserves to travel directly to the udder to sustain milk production [5]. Indeed, farmers are not only interested in high yielding but also in healthy, long (re-)producing animals. From the farmers’ perspective, production diseases are also a cost issue to which farmers can react differently [6]. For example, production diseases could be considered as undesired side effects of the production process, and factored in as associated failure costs. Alternatively, farmers could make use of investments and their work force to optimize living conditions and take preventive measures to reduce the occurrence of production disease and keep failure costs to a minimum. However, when striving for this alternative, farmers often have to face an associated increase in preventive costs.

The transition phase, defined as the period between three weeks before to three weeks after parturition [7], is the most challenging and critical period in relation to the dairy cow’s health status during the lactation cycle. Major physiological, nutritional, metabolic, and immunological changes occur within this time frame as the production cycle of the cow shifts from a gestational nonlactating state to the onset of copious milk synthesis and secretion [8,9]. Cows must adjust metabolically to the dramatic increase in energy and nutrient requirements needed to ensure milk production in the ensuing lactation. Gaps between nutrient demand and supply in the first weeks of the transition period can coincide with substantial variations in the diets’ nutrient content and in the daily dry matter intake (DMI), requiring comprehensive adaptation and regulation of the metabolism. Disturbances of one or multiple metabolic processes, related to the regulation of a certain metabolite in the body fluids, are known as metabolic disorders [10], and are a manifestation of the cow’s inability to cope with metabolic demands [11]. Clinical diseases most related to nutritional management are ketosis, milk fever, retained placenta, metritis, displaced abomasum, and lameness [12]. The colloquial associations among various metabolic stresses and their relationships to other diseases, particular infectious and inflammatory diseases of early lactation are interrelated and have become a central focus of the interest in metabolic diseases of dairy cattle [13].

Little is known about the limitations of dairy cows in their ability to adapt to and deal with variation and disturbances in their living conditions and the gap between nutrient supply and nutrient demand. Questions arising include: how to assess what is decisive for adaptation in the specific farm contexts. Making trade-offs between various nutritional and physiological traits requires further in-depth reflections on the background, the regulatory processes involved in metabolic pathways and the possible consequences. The complexity of the issue makes it necessary to consider it from different perspectives. The main objective of this paper is to reflect on the nutritional and physiological process of adaptation to the living conditions and on the prerequisites for a successful adaptation which avoids metabolic disorders in the transition period.

## 2. How does Adaptation Work?

To survive, dairy cows need to adapt to changing living conditions, challenged by varying gaps between individual needs and supply and by environmental changes or sudden and unforeseen disturbances. Stress response may be either non-adaptive, resulting in increased morbidity and mortality or adaptive, promoting overall fitness, emphasizing the ambivalent nature of stress. Not are only the stress factors manifold; the possible reactions of the animals to the same or to different stressors vary greatly. The adaptive responses of an individual animal may be specific to a given stressor, or general and independent of the type of stressor. Thus, adaptive success depends not only on the level and type of threat and on the responsiveness of the organism but primarily on the interactions between both.

Different terms are in use to describe the outcome of reactions in face of changes and disturbances. In this context, “robustness” can be seen as the property of being strong and healthy in constitution. The term means a kind of resistance to disturbance and irritation which prevents changes in a system’s composition occurring. Thus, robustness implies endurance but not necessarily adaptation which is a special manner of being tolerant to challenges by actively monitoring disturbances and compensating for their tendencies. The term “resilience” derives from materials’ sciences, meaning the power or ability to return to the original form, position, *etc.*, after being bent, compressed, or stretched. In contrast to “robustness”, changes within the system’s composition that occurred due to disturbances but were only temporary. In ecology, the term refers to “the ability of an ecosystem to return to its original state after being disturbed” [14]. With respect to agricultural systems, “resilience thinking focuses on enabling a system to cope with unexpected change and disturbance” [15]. In the case of dairy cows, this term might be used in describing the ability of an animal to regain a state within a normal physiological range and/or to recover from disorders and diseases. The term “adaptation” is used in biology in relation to how living beings adjust to their environments. In the face of changes and disturbances, reactions of organisms lead to changes in the composition of their system which remain in force and are more or less permanent. According to Di Paolo [16], adaptivity is a system’s capacity to regulate, according to the circumstances, its states and its relation to the environment with the result that, if the states are sufficiently close to the boundary of viability, (1) tendencies are distinguished and acted upon depending on whether the states will approach or recede from the boundary and, as a consequence; (2) tendencies of the first kind are moved closer to or transformed into tendencies of the second and so future states are prevented from reaching the boundary with an outward velocity. The specific rate of function for each organ, describing the specific range where it is able to operate, is specified by the regulatory system according to both environmental and internal conditions. A process becomes malfunctional when—due to the fact that its particular qualities limit its range of modulation—it is unable to do what the regulatory (sub)system “tells” it to do within the framework of a specific regime of self-maintenance. Diseases and disorders can be understood as negative bodily occurrences where a part of the organism fails to appropriately perform one of its biological functions [17]. Biological functions are interpreted as specific causal effects of a part that contributes to a complex web of mutual interactions, which, in turn, maintain the organization and, consequently, the part itself. Generally speaking, a well-adapted system is a set of interacting and interdependent entities, forming an integrated whole that together are able to respond to environmental changes or changes in the interacting parts.

Biological flexibility in the face of environmental demands is a fundamental prerequisite in regulatory physiological systems [18]. Moreover, regulation over a wide range of physiological and developmental conditions is required. Different concepts and terms have been coined in the past which refer to the necessary changes in regulatory processes. It is beyond the scope of this review to describe these concepts comprehensively. In short: In theoretical biology, an enduring tradition has placed heavy emphasis on the idea that biological systems employ what could be referred to as “self-determination” [19]. That is the capacity of a system’s constitutive organization to contribute to the determination and maintenance of its own conditions of existence through the effects of its activity. Following Bernard’s seminal work [20,21] during the first half of the 20th century self-determination was initially investigated as “homeostasis” [22] and mathematically expressed in terms of feedback loops by cybernetics [23,24]. For Cannon [25], homeostatic regulation reflects “a condition, which may vary but which is relatively constant”. A common example to explain homeostasis is the ability of the organism to maintain a glucose concentration in the blood within a certain range despite considerable changes in supply and demand of glucose and in turnover rates (“stability through constancy”). To keep the glucose concentration in the blood within a physiological range, changes in the organism have to take place, regulated primarily by neuroendocrine and endocrine secretions. The need to maintain the glucose concentration within a certain range is crucial also in the context of metabolic disorders and will be discussed in further detail below. Homeostasis is traditionally seen as belonging to short-term physiological adaptations. Selye [26] was searching for a concept beyond homeostasis which also described long-term adaptations, which he called “heterostasis” and defined it as “the establishment of a new steady state by exogenous stimulation of adaptive mechanisms through the development and maintenance of dormant defensive tissue reactions”. Waddington [27] suggested that homeostasis should be interpreted as “homeorhesis” which means stability of dynamics rather than stability of state. A crucial step in the theoretical development in understanding biological self-determination is the account put forward by Piaget [2]. His core idea was to integrate two inherent dimensions of biological systems into a single coherent picture: thermodynamic openness and organizational closure. In Piaget’s view, biological systems are self-determining because they are organized and they are organized because they realize closure. Organizational closure is the basis of many subsequent accounts of biological self-determination [28]. One of the best-known theories is the concept of “autopoiesis” [29]. In the context of dairy production, Bauman and Currie [30] refer to homeorhesis regarding the regulatory mechanisms of nutrient partitioning and physiological processes in different stages of life history, e.g., a lactating mammal is in a different homeostatic state from a pregnant female. A similar concept (“rheostasis”) involves the changes in regulation of state during circadian rhythms [31]. Knight *et al.* [32] used the terms “metabolic load” and “metabolic stress”. Metabolic load was defined as “the burden imposed by the synthesis and secretion of milk”, whereas metabolic stress was defined as “that amount of metabolic load which cannot be sustained, such that some energetic processes, including those that maintain general health, must be downregulated”.

The term “allostasis” was introduced by Sterling and Eyer [33] to refer to changing regulatory systems (“stability through change”). Allostasis can be considered as the process of maximizing fitness in the face of environmental change as well as unpredictable challenges. Regulatory mechanisms must change in order to maintain or achieve a state appropriate for the time of day or year and in response to disturbances. McEwen [34] developed the concept of allostasis further and coined the term “allostatic load” to describe potential permanent overburdening of homeostatic processes, e.g., by diseases. One can imagine allostatic load increasing due to the rising energetic cost of fueling regulatory processes. Accordingly, allostatic load is the sum of the energy required to maintain basic homeostasis and to acclimate to changing environmental conditions. Here, energy is assumed to be the sum of an individual’s requirements (including protein, essential fatty acids, vitamins, minerals, and so on) in any given state. McEwen and Wingfield [35] extended this idea further. They see allostasis as the process of adjusting morphology, physiology, and behavior to cope with allostatic load, while allostatic state is the hormonal state required to regulate predictable changes in an individual’s life cycle as well as unpredictable disturbances. Energetic demands on the individual can be further exacerbated by injury, disease, or social stress that could add cumulative allostatic load before all of the other energetic costs associated with the life cycle are considered.

Changes may occur as variation within a normal range. Predictable changes over time often emerge in an individual’s life cycle. However, unpredictable events, including many potential stressors, requiring immediate physiological and behavioral adjustments may also need to be dealt with. Disturbances can occur slowly or abruptly, e.g., as change in diet, climatic conditions (heat stress), pathogen pressure, competition with other individuals that may reduce access to trophic resources, injuries, diseases, and other challenges. Effects may be cumulative over hours or days and weeks, and are disruptive or lead to abnormal activity. Additionally, current infection and disease, social status, *etc.*, may influence how an individual animal goes about its routines and responds to unpredictable disturbances. In the case of dairy cows, challenges to their adaptation can be differentiated as follows:

- A group of animals or the dairy herd is exposed to the same feeding and housing conditions, although individual cows can vary considerably in their needs, due to differences in live weight, milk yield, age, or social rank. The bigger the individual gap between existing and optimum living conditions, the more cows are challenged to adapt.
- Unforeseen and abrupt changes in the animal’s environment, e.g., from chemical and physical factors generally concern the whole group of animals. However, individual animals may vary in their capability to react to these changes for various reasons.
- Cows within a herd face individual challenges due to disturbances from ranking conflicts or due to handicaps caused by clinical and subclinical diseases or injuries.
- Cows which simultaneously undergo changes in the environment and within the organism face the biggest challenges; *i.e.*, in the transition period. Considerable morphological, physiological, hormonal, and behavioral changes occur which serve in part to prepare the body for the period leading up to birth and for calving itself and also to get the cow’s metabolism ready for impending lactation. These adaptations consist of mid-pregnancy anabolism followed by a significant shift to catabolism in late pregnancy and a dramatic catabolic shift in early lactation. At the same time, cows are offered a different diet than in their dry period and are often moved to different living conditions.

This paper focusses on the possible implications of metabolic stress caused by changes and disturbance in nutrition on a metabolic level. The current knowledge about the relationship between metabolic disorders and production diseases is outlined in brief before the above issue is further explored with reference to variation on different process levels.

## 3. Metabolic Disorders and Production Diseases

Desirable outcomes in farm management are cows that are successful in adapting metabolically to challenges in the transition period with minimal to no disease events, reduced avoidable culls, and efficient productive and reproductive performance. However, the reality often differs greatly. Negative side effects of the production process are obvious. They have been intensively discussed since the first Intl. Conference on Production Diseases in Farm Animals in the year 1968 which has occurred periodically ever since [36]. Traditionally the term “production disease” was regarded as encompassing the significant metabolic disorders of dairy cows (hypocalcaemia, hypomagnesaemia, and ketosis), but has been broadened in the course of time to include conditions such as a retained placenta, displacement of the abomasum, metritis, and laminitis [11]. With the increase of milk production, diagnostic method sensitivity as well as recognition of subclinical disease also increased. Dairy farms continue to be plagued by high prevalence of cow disorders, adversely affecting productivity, reproduction, and animal health and welfare [37,38]. Much intensive research has been conducted to address nutritional requirements, physiological adaptations, and metabolic associations with periparturient disease of cows in the transition stage. According to LeBlanc [39], dairy production is challenged by the fact that 30% to 50% of dairy cows are affected by some form of metabolic or infectious disease around the time of calving. Compiled periparturient disease incidence rates are presented in Table 1. According to Van Saun and Sniffen [40], most in the dairy industry still believe that there are tremendous opportunities to improve transition cow health and reproductive performance without compromising milk production, yet the solution is not clearly evident.

**Table 1:** Compiled periparturient prevalence of metabolic disorders from various published studies according to Van Saun and Sniffen [40].

Disease	Median Incidence Risk (%)	Range of Incidence Risk (%)
Hypocalcemia	6.5	0.3–22
Subclinical hypocalcemia	2.2	8–54
Retained fetal membranes	8.6	1.3–39.2
Metritis	10.1	2–37
Subclinical metritis	53	37–74
Ketosis	4.8	1.3–18.3
Subclinical ketosis	43	26–55
Lameness	7.0	1.8–30
Clinical mastitis	14.2	1.7–54.6
Subclinical mastitis	30	15–60

In contrast to the comprehensive data sets on the performance traits of dairy cows, the monitoring of metabolic disorders is often restricted to data from single scientific investigations. Unlike clinical diseases, subclinical metabolic disorders are even more difficult to detect; they require additional diagnostic tests, and obtaining a complete picture is not always possible. This refers not only to the severity of subclinical disorders but also to impacts which metabolic disorders might have on the development of infectious diseases and fertility disorders or mortality and culling rates, respectively. In the past, comparably few studies have been carried out to investigate relationships across a broad spectrum of health problems in relation to milk production. For example, in the case of subclinical ketosis, the reported prevalence for hyperketonemia in the first two months of lactation ranged widely from 8.9% to 34% in various studies [41,42]. Comparing current incidence rates of disease with those from past years is not helpful as records are not necessarily reliable, and cows who produce more milk than their herdmates are not automatically at increased risk of developing disorders [43]. According to Mulligan and Doherty [11] the hypothesis that high yielding cows automatically have higher levels of production diseases is likely to be as false as the hypothesis that lower yielding cows suffer from lower levels of production disease.

In early lactation, cows are in a stage of negative energy balance (NEB) caused by a rapid increase in the demand for nutrients to support milk production which exceed the increase in food intake. This results in lower blood glucose and mobilization of body reserves to provide additional energy. These processes are accompanied by elevated blood concentrations of non-esterified fatty acids (NEFA) and ß-hydroxybutyrate (BHBA) and decreased levels of calcium and phosphorus. According to Drackley [7], many health disorders in dairy cattle are attributed to the process of uncontrolled lipid mobilization in response to excessive NEB in early lactation. All of these metabolic changes increase the risk for ketosis, hepatic lipidosis, hypocalcemia, and infectious diseases, such as mastitis and metritis [44,45,46,47]. However, there is considerable variation in the plasma concentration of substrates during the day (diurnal variation) and over a period of days in early lactation. For example, plasma BHBA concentration of cows with clinical signs of metabolic disorders, generally used as an indicator for ketosis, differs widely as does the maximum plasma concentration measured in cows that did not develop clinical ketosis in the first six lactation weeks [48]. Changes in the concentrations of metabolites and hormones during the postpartum period differ remarkably amongst animals kept under similar and highly standardized conditions on a research farm, indicating that the ability to cope with metabolic stress varies considerably between individual cows [49].

Sordillo and Raphael [9] addressed the possible connections between fat mobilization and dysfunctional inflammation responses that may contribute to increased morbidity and mortality in the transition phase. While an efficient inflammatory response eliminates the invading pathogen, restores immune homeostasis, and returns tissues back to normal function and morphology, many aspects of the bovine immune system are compromised around the time of calving, especially on the level of inflammatory responses [50]. The authors conclude that the delicate balance between a sufficient inflammatory response needed for optimal pathogen clearance and the prompt return to immune homeostasis is often lost during the transition period. Immune suppression in the periparturient dairy cow is a commonly observed phenomenon and has been linked to poor metabolic status and negative energy balance [51,52]. In cows suffering severe NEB, inflammation immune genes are upregulated [53], whereas genes involved in the acquired immune responses are downregulated [54]. The mechanisms of impairment of immune defense in the transition period have been described in further detail with regard to the mammary gland [55] and the uterus [56,57]. Cows with severe metritis ate less than healthy cows in the two to three weeks preceding the clinical signs of metritis [58]. Lower feed intake is associated with increased circulating concentrations of non-esterified fatty acids (NEFA) which may directly [59] or indirectly [60] inhibit neutrophil function. Due to both high metabolic demands and pathogen challenges, cattle routinely experience substantial oxidative stress in early lactation too [61], which also contributes to a pro-inflammatory state that impairs immune defense [62]. The authors highlight that a major underlying factor in the development of transition cow disorders is metabolic stress, which occurs when cows fail to adapt physiologically to an increase in nutrient requirements needed for parturition and the onset of milk synthesis and secretion. The combined effects of altered nutrient metabolism, dysfunctional inflammatory responses, and oxidative stress can form destructive feedback loops that exacerbate metabolic stress and cause health disorders.

The role of inflammation-associated pathways in adaptation processes is not yet fully understood. The findings by Farney *et al.* [63] suggest that inflammation induced insulin resistance is, in some cases, an adaptive rather than pathological, phenomenon. Some degree of inflammation might even be required for successful adaptation. On the other hand, increased concentrations of analytes associated with stress and inflammation during the periparturient period are associated with lower milk yield and compromised reproductive performance later in lactation [64]. An underlying factor of dysfunctional inflammatory responses is the overproduction of reactive oxygen species, leading to oxidative stress [65]. While free radicals are essential for physiological processes, imbalanced or excessive free radical production plays a key role in the pathogenesis of diseases. Oxidative stress has been identified as a link between nutrient metabolism and inflammation during the transition period [62].

A close relationship between NEB and fertility disorders has been revealed and reviewed in many studies [5,66,67,68]. Increased locomotive problems are also associated with longer and more extreme periods of negative energy balance [69]. As a consequence, individual production diseases of the dairy cow should not be considered in isolation. For example, ketosis, fatty liver, retained placenta, hypocalcaemia, metritis, and displaced abomasum may be all interrelated [11]. Primary causes and the confounding factors which contribute to the development of metabolic disorders are manifold and vary considerably between farms. These factors are not only related to the feeding regime but are complicated by various management issues. Irrespective of average milk yields, some farms do well whilst others fail quite markedly in reducing clinical and subclinical problems. Vastly different nutrition and management programs produce equally good or equally poor success in relation to metabolic disorders [7]. However, little is known about the relationships between factors like body condition, feed intake, and postpartum health and performance, each varying so widely among individual cows.

## 4. Challenges Due to Varying Living Conditions

Dairy cows live in environments that change over time. Changes that affect their self-determination and self-preservation are particularly relevant. In the first place, reactions of animals to changes in the environment are thus self-referential [70]. Cows with different and varying needs have to adapt to more or less identical living conditions. Total mixed rations are generally formulated according to the average performance of a herd or a group of farm animals. As such, rations are not adequately adapted to the requirements of individual cows within a feeding group or herd. Group size, grouping strategy, and group feeding behavior have a considerable impact on the competition between animals for feed and space and thus on feed intake [71]. Competition is perceived differently by the animals, depending to a high degree on the social rank they hold. Cows in uncomfortable conditions are less likely to use their potential for adaptation.

Living conditions are quite complex, encompassing various nutritional, hygienic, and housing spheres as well as social environment. Each sphere includes a multitude of influencing factors, differing considerably in their impact on the animals. Furthermore, they change in varying degrees over time. While climatic conditions can change quite quickly, the dimensions of space allowance, lying areas, and bunk space are more or less given conditions in a stable. Also feeding regimes on a farm might appear steady, but nevertheless can evolve more or less extended modifications in the nutrient and energy supply. Diets are composed of various ingredients, which differ not only between proportion of carbohydrates, proteins, fat, minerals, and trace elements but vary considerably in the solubility of the different carbohydrate and protein fractions and correspondingly in the availability for the animals within the digestive tract [72]. Charges of roughage or concentrate as part of the total ration differ widely in their composition. Furthermore, the proportion of single components can vary substantially due to imprecision in allocation or in mixing procedure. A comprehensive overview of the countless nutritional factors on the farm level that cause variation between and within dairy farms has recently been provided by Oetzel [12].

The amount of daily feed intake is a resultant of interactions between the composition of the diet itself, the environment in which a diet is offered and various intrinsic processes [73,74]. Feed and energy intake can change dramatically in response to changes in diet composition or metabolic state. Such changes are poorly predicted by traditional models of feed intake regulation [75]. Variation in feed intake does not only refer to the daily dry matter intake (DMI). The same DMI per day can be achieved by different frequencies and durations of eating time and meal sizes, respectively. Thus, it is not surprising that a large inter- and intra-individual variation of feed intake is observed in farm practice [76]. Even if a balanced diet following the defined average intake for a given feeding group is provided, a large variation in DMI within a group results.

When provided with free access to high nutrient dense feed, dry cows do not regulate and restrict their intake to meet their requirements and thus will over-consume energy. Dann [77] recorded intakes by dry cows that were up to 60% in excess of their requirements when fed ad libitum, with a large variation between cows. Lactating cows, provided with a suitable ration will readily consume in excess of 3.5% of their body weight as feed DM daily. This equates to between 22 and 25 kg DM/day, yet in many situations, intakes often fail to achieve even 20 kg DM/day [74]. Feed intake is depressed when highly fibrous, bulky food is fed or diets are deficient in essential components such as protein or feed access is restricted by time, bunk space, or competition, and when environmental temperatures and humidity increase and create heat stress [71,78,79]. Bunk space is most critical in farms where feed availability is limited or where overcrowding occurs. Fresh cows seem to be more sensitive to behavioral competition and intimidation, which results in altered feeding behaviors where feed bunk space is inadequate [79]. As the social hierarchy is more frequently altered, observational studies suggest a decline in feed intake, with observed greater disease prevalence [80]. The reasons for the large variation in DMI, even under standardized living conditions, are manifold, reliant on influencing factors both outside and inside the organism. Interactions between the different influencing factors create a virtually unlimited variety of combinations. What makes it nearly impossible to assess the extent of the impacts of feeding, social, and housing conditions is the fact that impacts do not emerge separately from one other. Living conditions have impacts and are challenging the animals as a whole. On the other hand, a reduction in feed intake is generally the first observed sign in cows developing metabolic disorders, particularly clinical ketosis [81]. Thus, the question always remains open, whether the feed intakes are primarily caused by impending disease, or whether the feeding patterns are induced and then result in increased disease risk?

## 5. Variation in Metabolism

Digestion by ruminants is the net result of a sequence of processes that occur in different segments of the gastrointestinal tract (GIT). These processes are characterized by degradation, passage, and absorption; they compete directly with one other. The sequence includes fermentation of dietary components by microbes in the rumen, acid hydrolysis, and degradation by the host animal’s enzymes in the abomasum and small intestine, and secondary fermentation in the cecum and large intestine. The passage rate determines the time that feed is retained in various compartments of the GIT for digestive action and affects the nature of end-products absorbed [82]. First of all, the feed reaches the rumen, which is a large fermentation chamber, containing a complex microbial ecosystem that works in a dynamic, symbiotic relationship with the host to convert feed into energy and protein. This microbial ecosystem consists of bacteria, protozoa, archaea, fungi, and bacteriophages and is highly responsive to dietary changes [83,84]. The diverse synergies and antagonism of ruminal microbes allow ruminants to efficiently use a range of feeds. Individual cows seem to have a rumen ecosystem comprising a core rumen microbiome that adapts to different feed substrates [85]. Thus, the composition of the rumen microbiome reflects the diet, feed additives, health, age, and seasonal conditions [86,87].

To understand the dynamics of ruminal fermentation, it is necessary to understand the interactions between the rate of passage of feed through the rumen and the rate of digestion of feed in the rumen. The competing rates of fermentation and passage from the rumen dictate how much of a fermentable fraction is fermented in the rumen and how much passes undigested to the abomasum. Passage from the rumen involves mixing, disruption, and comminution of various components. The rumen operates as a partially stirring, continuous-flow reactor and different sub-compartments with different flow characteristics may be distinguished: liquid, escaping particles, and retained particles [88]. In contrast to the extensive amount of data available on substrate degradation rates, the availability of data on ruminal retention times is relatively scarce. Production and absorption of volatile fatty acids (VFA) constitute dynamic processes resulting in large postprandial variations in ruminal concentrations. Close relationships exist between production rates, the ruminal concentrations and the fluxes of VFA across portal drained viscera. The fractional ruminal VFA absorption rates increase with chain length and decrease with pH [89,90]. Diets higher in non-fiber carbohydrates (NFC) content promote the development of ruminal papillae for adequate absorption of volatile fatty acids produced during ruminal fermentation [91].

Effects of pH on ruminal VFA concentrations and kinetics of VFA absorption by the rumen wall differ considerably [89]. While removal of volatile fatty acids (VFA) from the rumen by absorption through the rumen wall are major processes that influence ruminal pH, VFA and lactic acid can build up in the rumen and reduce ruminal pH. In addition, the buffering capacity of rumen fluid is variable and is generally assumed to depend primarily on bicarbonate. Bicarbonate dependent absorption is not just a primary absorption pathway of VFA but bicarbonate can also be secreted at a capacity equal to that from saliva, thus removing protons from the rumen by neutralization. In addition, the inherent buffering capacity of the diet is involved in pH regulation, largely explained by the cation exchange capacity of feedstuffs. Low ruminal pH for prolonged periods each day can affect feed intake, microbial metabolism, and feed digestion, and has also been related to inflammation, diarrhea, and milk fat depression [92]. In face of the various impact factors, empirical models to predict ruminal pH have so far had limited success. In a recent study [93], large differences in reticulum rumen pH development were observed postpartum between individual animals when assessed continuously with an indwelling and wireless monitoring system. Considerable differences occurred despite the fact that all cows were kept under the same conditions and received the same diet before and after parturition. The results indicate that rumen pH-values are not exclusively influenced by the diet and it is thus hard to predict using experimental models; at least not on the level of the individual animal.

Fermentation rates of NDF decrease from relatively acidic to almost neutral pH [94]. The rate of fermentation of water-soluble carbohydrates and starch is, among others, affected by the amount of ruminal degradation of dietary protein [95]. Increases in the amount of ruminal degradation of dietary protein relative to readily available carbohydrates result in reduced amounts of carbohydrate stored as glycogen by the microbes, which means that the carbohydrate from the diet is fermented soon rather than stored. Another effect of increasing ruminal degradation of dietary protein relative to available carbohydrate is an increase in yield of microbial protein per unit of carbohydrate [96]. The competing rate of fermentation of starch has been reported to increase as the amount of starch in the diet increases [97]. The quantity of amino acids available to dairy cows is, among others, dependent on the ruminal degradation of dietary protein, the capacity of the rumen to use non-protein nitrogen sources for the synthesis of microbial protein, the degree to and efficiency with which ruminal degradation of dietary protein can be synthesized into microbial protein, the variation in value of different proteins to the animal that escape ruminal degradation, and endogenous protein losses [78]. Thus, there is considerable day to day variation in the efficiency of the conversion of metabolizable protein (MP) to net protein and estimates of microbial nitrogen outflow from the rumen [98]. To sum up, substantial day to day variations in digestion and fermentation processes in dairy cows cause considerable variations in the relative quantities and supply of essential nutritional elements from the intermediate metabolic processes. This supply variation represents a real challenge for the metabolism in the face of demands for milk production and maintenance.

## 6. Discrepancy between Nutrient Demand and Supply

The average milk yield per cow has increased considerably over the last decades, primarily as the result of genetic selection. Additionally, increase in production has been supported by the improvement in environmental factors such as feed composition, feeding strategies, housing conditions, and farm management. Breeding programs have become quite successful because of the comparatively high accuracy of breeding value estimation, the moderate to high heritability of most production traits, and the use of large and fast databases containing production records of many animals and their genetic relationships [38]. The authors emphasize that increase in milk production is twice as heritable as feed intake, leading to the consequences that extent and duration of negative energy balance (NEB) has considerably increased over the last decades. During lactation, an increase in milk yield with a peak between the fourth and sixth week postpartum is functionally based on a marked increase of cell differentiation and tissue hypertrophy in the udder [99]. The number of vital mammary epithelial cells control the initial conditions for the amount of milk produced and the amount of glucose needed for the production and secretion of lactose [100]. Treating cows with bovine somatotropin could help to improve persistence of milk production because it prevents, among other things, an increase of the enzyme plasmin in the milk and thus the involution of udder epithelia. This emphasizes that the power drop in milk performance is an important programmed, endocrine and autocrine regulated process [101].

Mammary epithelial cells have an incredible level of organization and a remarkable ability to convert circulating nutrients into milk components. Cows with a high genetic performance capacity for milk production are characterized by the ability to perform intensive gluconeogenesis and partitioning of the glucose into the udder. According to Bauman *et al.* [102], the productivity of this biological factory is extensive and in terms of the use of nutrients and energy, the cow should be viewed as an “appendage to the mammary gland” rather than *vice versa*. To produce milk, the udder requires a corresponding amount of nutrients, particularly glucose, from the intermediate metabolism which correlates greatly with the amount of milk secreted. Hepatic glucose production is essential for the ultimate production of lactose which controls milk volume. The demand for glucose varies in connection with the intra- and inter-individual variation of daily milk yields. In contrast, fat is the most variable milk component, predominantly affected by nutrition. Being the major energy component in milk, milk fat percentage and fat yield can be reduced by 50% or more with little or no change in the milk yield or other milk components [102]. Milk fat is composed of fatty acids of varying chain length and degree of saturation. These are derived in almost equal manner from the uptake of circulating preformed fatty acids and the synthesis of new (de novo) fatty acids in the mammary gland. Gardner *et al.* [103] compared starch and fat supplemented rations fed to multiparous cows and noted a 4 kg/day improvement in milk yield for those fed enhanced fat levels. Fat supplement reduced milk protein content by 8% whilst milk fat levels were unaffected, but overall milk fat and protein output was increased by 9%. Computing their energy balances showed that the fat supplemented diet increased tissue mobilization.

In a study by van Knegsel *et al.* [104] cows were fed on an isocaloric and isonitrogenous basis either a mainly glucogenic (by-pass starch) or a mainly lipogenic diet. Results showed that the glucogenic diet stimulated energy partitioning towards body reserves in early lactation. Cows fed the glucogenic diet had lower NEB and reduced fat mobilization, which led to milk fat depression and less energy partitioned to milk. Thus, energy partitioning between milk and body tissue can be altered considerably by diets that differ in lipogenic and glucogenic nutrient content. High yielding cows fed with a diet enriched with crude protein (19.9%) produced an extra 5 kg milk/day of similar fat but lower protein contents in comparison to a diet with 16.7% crude protein, while ME intakes showed no treatment differences [105]. The author concluded that the extra production of milk and milk solids in high protein diets had been achieved by increased mobilization or reduced depletion of body condition. Due to a sudden increase of nutrient requirements for milk production postpartum at a time when dry matter intake and nutrient supply lag behind, nearly every high yielding cow faces the challenge of negative energy balance. NEB describes the portion of the total energy requirements of the dairy cow for maintenance and milk performance which is not covered by nutrient supply from feed intake [106]. This portion has to be provided by the organism in making use of depot nutrients to gain a balance between output and input levels. Increases in genetic merit for milk yield go together with increases in feed intake, but the latter does not fully compensate for the extra energy demands during early lactation, resulting in a more or less extended negative energy balance and increased mobilization of body reserves [107,108].

Calculated energy balance is typically mostly negative within the first 12 days postpartum [106]. Differences among cows in nadir and total energy deficits are large. Over the course of 122 lactations, mean values of total energy deficits during early lactation amounted to 1451 MJ NE_L_ with a standard deviation of 1062 MJ NE_L_. The postpartum interval to nadir of the estimated energy balance averaged to 48 ± 29 days. First lactation cows showed a smaller energy deficit in early lactation than older cows. Moreover, cows differ considerably with regard to the partitioning of energy between different physiological systems. Thus, cows with similar energy intakes and expenditures via the milk may actually experience differences in the burden of NEB. This does not only refer to genetic make-up (e.g., high v. low genetic merit) or the stage of lactation, but varies greatly between individuals of the same genotype or in the same stage of lactation [109]. This makes predicting the dynamic of nutrient partitioning in a generalizable way (*i.e.*, across physiological stages and genotypes) more challenging.

Furthermore, emotional stress affects NEB via feed intake and glucose and free fatty acid metabolism, mainly via the sympathetic nervous system, and, to a lesser extent, via HPA axis [110,111]. Glucose is the main substrate covering energy expenditure during activities which contribute to acute emotional stress, whereas fatty acids are the main substrate which covers energy expenditure during prolonged demands. Inflammation postpartum, coupled with an increased cellular metabolism, accelerates immune gene expression while mitochondrial uncoupling further increases energy requirements and exacerbates negative energy status [112]. While numerous studies revealed that NEB can be responsible for various health disorders such as ketosis and infectious diseases [51], health problems (e.g., digestive or locomotive problems) can also be a trigger for NEB and may affect the NEB negatively in early lactating cows.

## 7. Metabolic Adaptation

For a successful transition from late pregnancy into lactation, a cow needs to carefully coordinate metabolism across multiple tissue layers to provide sufficient nutrients and energy to support productive needs. Daily requirements for glucose, amino acids, fatty acids, and calcium for an early lactation cow are more than 2.7, 2.0, 4.5, and 6.8 times greater, respectively, than those needed for pregnancy [8]. These differences represent changes in nutrient requirements over a short period of only one to two weeks, highlighting the tremendous metabolic alterations necessary to adequately support lactation. This means that it is understandable that a cow with an inadequate ability to adapt metabolically to this transition can be the root of most postpartum diseases. Imbalance in energy and nutrient supply of high yielding cows in early lactation is inescapable. The intensity of the imbalance is influenced by both, the level of milk yield and the degree of endogenous energy provision. Levels required from the nutrient stores to compensate deficits can be theoretically calculated as the discrepancy between the total requirements and the resources deriving from daily feed intake. Generally, increase in genetic merit for milk yield is the most pronounced determinant of increased body condition loss and allostatic load [109,113]. In a study by Sutter and Beever [114], tissue mobilized over the first eight weeks of lactation was estimated to support the production of 300 L milk, from a total recorded production of 1820 L milk. Total milk production in week one was 33.8 L/day, of which 10.8 L/day (32%) were estimated to be derived from mobilized tissue. High-yielding dairy cows have been genetically selected to partition even more energy reserves into milk production with the effect that reliance on body reserves has dramatically increased [115]. Even in the case of higher dietary intake, the increased input will primarily result in greater milk production, while a similar energy imbalance remains, with no beneficial effects on body condition and reserves at all [116].

First and foremost, the mobilization requires well-functioning regulatory capacities that enable an efficient exploitation of resources, orchestrating a release of nutrients matching the requirements to a high degree and dealing with possible bottlenecks in metabolic pathways. The goal of regulation is not to preserve the constancy of the internal milieu; rather, it is to continually adjust the milieu to promote survival. The question is how the organism assesses and deals with the differences on a short- and long-term basis respectively; allowing the mobilization process to become more efficient. Furthermore, there is a question of whether the organism can rely on the regulatory opportunities available to it to prevent exhaustion due to overwhelming demands. The process of adaptation to NEB involves metabolic interrelationships between the energy storage site in the adipose tissue and skeletal muscle, the fuel processing site in the liver and the energy use sites in the mammary gland, placenta, fetus, and other tissues of the body. An increased competition for resources arises within the organism in the light of limited availability of resources. Health problems occur when these interrelationships and the adaptation process do not function appropriately to allow the cow to get through the period of NEB in early lactation.

Adaptation consists in the first place of physiological shifts in the use and conservation of body fuels: carbohydrates, amino acids and fats. The major mechanism involved is shifting the body’s fuel supply away from glucose and toward the use of lipid energy sources. Lactation has an obligatory requirement for glucose that cannot be met by other fuels. Substrate-mediated events initiated by a decline in blood glucose concentration result in mobilization of non-esterified fatty acids (NEFA), which in turn results in several actions that stabilize or increase blood glucose. Besides NEFA, glycerol and proinflammatory cytokines, (TNF, IL-6) are also released [117]. Neuroendocrine and endocrine mediators are essential in the ability to adapt to NEB because fuel-mediated adaptation is not sensitive enough to function independently. Thus, endocrine actions primarily serve to control the rate of fuel metabolism. Additionally, the presence and number of specific receptors as well as their affinity play a decisive role in the mechanisms of metabolic regulation [118]. Initiation and persistence of a nutritional imbalance in early lactation in favor of the provision of external and internal nutrients for milk production is managed by simultaneous changes in the concentration of insulin, glucagon and growth hormone [119]. Low molar quotients of hormone concentrations ensure a maximum nutrient flux and nutrient uptake towards the udder. Growth hormone (GH) increases before calving [120]. High GH concentrations not only stimulate milk production but also provoke liver gluconeogenesis and lipolysis in adipocytes. Despite increased gluconeogenesis, blood glucose levels are low because of the drain of glucose to the udder. The mobilized NEFAs serve as an alternative energy source for other tissues to preserve glucose, which is used preferentially by the mammary gland to form lactose [121]. Hypoinsulinaemia promotes gluconeogenesis in the liver and acts as a massive lipolytic trigger. The udder benefits because it does not need insulin to facilitate glucose uptake into cells by the glucose-transport molecules [122]. Furthermore, low insulin concentrations uncouple the growth hormone (GH)-insulin like growth factor (IGF-I) axis in the liver because of down regulation of GH 1A receptors [123]. The resulting high blood NEFA and GH concentrations antagonize insulin action and create a further state of peripheral insulin resistance in liver, muscles, and adipose tissues [124]. In this way even more glucose is conserved to be available for lactose synthesis. Fat has been described as the “master regulator in the development of systemic insulin resistance” with good reasons [125].

Problems can occur in several steps in the adaptation process, particularly involving the adipose tissue and the liver. In the dairy cow, adipose tissue lipid accumulates during pregnancy, and catabolism begins prior to parturition and increases dramatically afterward. The duration and magnitudes of these adaptations depend on milk energy secretion, net energy intake, genotype, and endocrine environment [126]. Adipose tissue lipid synthesis is decreased and lipolysis is increased in early lactation. The magnitude and duration of these adaptations are increased in animals either consuming less energy or producing more milk. Adipose tissue is more sensitive to catecholamines in early and in midlactation and in animals with higher production. Adaptive responses of adipose tissue turn out to be inappropriate because of alterations in adipose sensitivity [9]. As adipose sensitivity increases, so does the NEFA response to a given stimulus such as catecholamine. When adipose tissue is highly sensitive, antilipolytic feedback responses may be blunted, releasing adipose tissue from metabolic and endocrine controls and allowing the rate of NEFA mobilization to go unchecked [124]. Different mechanisms lead to increased adipose sensitivity in obese cows, resulting in an interruption of homeostatic mechanisms regulating adipose mobilization. For example, high genetic merit in dairy cows is associated with increased adipose sensitivity [127,128] and a decline in circulating insulin levels [66]. During NEB, the GH-IGF axis uncouples due to a downregulation in liver GHR and this is associated with a reduction in circulating IGF-I and elevated GH concentrations, providing an endocrine environment that promotes the direct action of GH on lipolysis and gluconeogenesis in early lactation [129]. The adipose levels in a cow are also a critical factor affecting adipose sensitivity. Studies conducted by Rukkwamsuk *et al.* [130,131] revealed that cows on a high-energy diet ad libitum throughout the dry period had adipose tissue that was less responsive *in vitro* to the antilipolytic effects of glucose and ketone bodies compared with restricted-fed controls. This reduces the effects of fuel-induced, autoregulatory negative feedback signals on lipolysis, allowing increased NEFA mobilization in obese animals. In response to NEB, over conditioned cows mobilize more body fat than thin cows and subsequently are prone to develop metabolic disorders [132]. Obese cows have less effective insulin feedback regulation in the adaptive response to NEB and greater adipose sensitivity and NEFA mobilization compared to leaner cows [133]. This can result in inappropriately high blood NEFA concentrations that overwhelm the capacity of the liver for fuel processing and interconversion.

Recent findings revealed the effect of dietary level of energy during the dry period on feed intake, energy balance, milk production, and composition [134]. Cows being fed a diet exceeding energy requirements during the whole dry period showed an increased incidence of hyperketonemia while a controlled-energy dry period feeding approach was successful in minimizing the degree of negative energy balance postpartum as well as hyperketonemia. Several studies have shown that the energy level and composition of the pre-partum diet also have an impact on lipid metabolism, liver function, and the overall health of the cow post-partum [135,136]. A transient decline in high density lipoproteins and loss of this molecule’s anti-inflammatory and antioxidant actions may account for a portion of the increased oxidative stress recognized to alter immune function in transition cows [9,137]. Besides fat, protein also plays an important role in the provision of glucose. Absorbed and mobilized amino acids from the muscles are partitioned to either gluconeogenesis or ketogenesis (oxidized for energy), milk protein or body (and conceptus) proteins. Although utilization of propionate for gluconeogenesis is extensive, amino acids have the potential to increase in importance as gluconeogenetic substrates when glucose demand is substantially increased [138]. Van der Drift *et al.* [48] observed large variation in the onset and duration of periparturient fat and protein mobilization. Cows mobilized in total 35% ± 26% of back fat thickness and 18% ± 9% of longissimus muscle thickness when kept under similar conditions at an experimental farm. Ketone body production was restricted in cows that mobilized more muscle protein relative to fat tissue after parturition. The relative partitioning amongst these pools depends on the mix of nutrients entering the body, the availability or need to replenish body tissue stores, and other physiological influences on the cow (stage of lactation, phenotypic, and genetic capacity for milk production, environmental conditions, age, growth stage, previous nutrition).

The hepatic response to NEB is to produce glucose and ketone bodies and take up large amounts of NEFAs from the blood for mobilization as other forms of energy. Apart from the activity of hormones, metabolic regulatory events in the liver are directed primarily by the level of metabolites, either in excess (e.g., free fatty acids) or shortage of supply (e.g., glucose) [139]. Reynolds *et al.* [140] reported major changes in the hepatic flux of NEFA as cows progressed from a pregnant state into lactation. Measured NEFA flux at day 19 before calving was equivalent to approximately 1 mol palmitate equivalent/day and a similar level was recorded at day nine before calving. In contrast, by 11 days post-calving, NEFA flux increased to 5.5 mol palmitate/day and whilst the level declined as lactation progressed, it was still almost twice the pre-calving baseline at lactation day 83. Maladaptive responses in the liver can arise at several points in the metabolic processes. Comprehensive reviews on the pathophysiological basis of ruminant adaptation to NEB and the development of ketosis and fatty liver is provided by Herdt [141], Drackley *et al.* [47] and Ingvartsen [112]. In short: Type I ketosis occurs when the demand for glucose outstrips the capacity of the liver for gluconeogenesis. Pathways for gluconeogenesis might be at the height of stimulation, but the supply of glucose precursors is insufficient to permit maximal glucose production. Thus, glucose production is limited by substrate supply in the first place.

Type II ketosis occurs when large amounts of NEFAs are delivered to the liver, but gluconeogenesis and ketogenesis are not at the height of stimulation. Mitochondrial uptake of NEFA is not as active as in type I ketosis. NEFAs not used for ketone body synthesis are esterified in the cytosol, forming triglyceride. Transport of triglycerides from the liver to other tissues requires the synthesis of very low-density lipoproteins (VLDL). Secretion may be near disabled when blood NEFA concentrations are high, leading to an accumulation of triglycerides in the liver. As the degree of fatty infiltration increases, normal functions of the liver are adversely affected. For example, the development of a fatty liver can interfere with hepatic gluconeogenesis capacity [142,143]. The ensuing hypoglycemia can lead to further adipose mobilization, creating a destructive spiral of events leading to fatty liver of increasing severity [141]. Fat infiltration also impairs the ability of the liver to detoxify ammonia to urea [144]. Ammonia decreases the ability of the liver to convert propionate to glucose [138]. Fatty liver and type II ketosis are usually seen clinically shortly after parturition, well before peak milk production. This reflects the association with adipose sensitivity [145]. Fat accumulation in the liver begins to occur in the last weeks of gestation, as adipose sensitivity increases. Factors influencing adipose sensitivity and energy balance in the late dry period are important concerns in the prevention of fatty liver and type II ketosis.

## 8. Hierarchically Organized and Nested Systems

The previous explanations followed the processes during the flux of nutrients from the environment through the digestive tract, and processes within the intermediate metabolism on different scales. As stated above, transformation processes are influenced by countless variables on each scale. Changes in perspectives from a top down to a bottom up approach and *vice versa* might help to improve our understanding of what restricts cows’ metabolic capabilities to adapt to nutritional changes and to grasp metabolism with a genuinely systemic approach. Ideally metabolism should be expressed mathematically, but this is made difficult by the fact that its elements are simultaneously both variables and functions [146]. Metabolism is the inherent process of living organisms to obtain nutrients and energy from the feed ingested for self-referential purposes. Feed is made up of substrates (proteins, carbohydrates, and fats, *etc.*), degraded by enzymes in the digestive system into absorbent nutrients and fuel. The body can use the nutrients and the fuel right away, or it can store them in body tissues, such as body fat and muscles. A metabolic disorder occurs when abnormal chemical reactions in the body disrupt or impair the process of utilization. When this happens, the animal might have too much of some substrates, too little of other ones, or too much and too little simultaneously so that proper function is disturbed. A metabolic disorder can develop also when some organs become diseased or do not function properly. Thus metabolic disorders can be divided to structural and functional components. A structural component is represented by the arrangement and configuration of compartments (organs, tissues, cells). Details of a structure can vary considerably between animals, e.g., in relation to the proportional size of sub-systems and their functionality. For example, the large variation in number and form of villi in the rumen, and the condition of other semipermeable borders which have a considerable effect on the flow of nutrients between the compartments [147], illustrate structural variability. Functional components contribute to self-regulatory mechanisms, responsible for the stability of the body’s internal environment [148]. Their effects can be regarded as dynamic constants, including blood metabolites, osmotic pressure, or pH amongst others. However, metabolic and external influences cause permanent or periodic changes in parameters of the internal environment. Therefore, the mode of activity and parameters of results of metabolic functional systems change consecutively. Self-regulation of functional systems is the mechanism which maintains these parameters at optimal levels.

**Figure 1:**
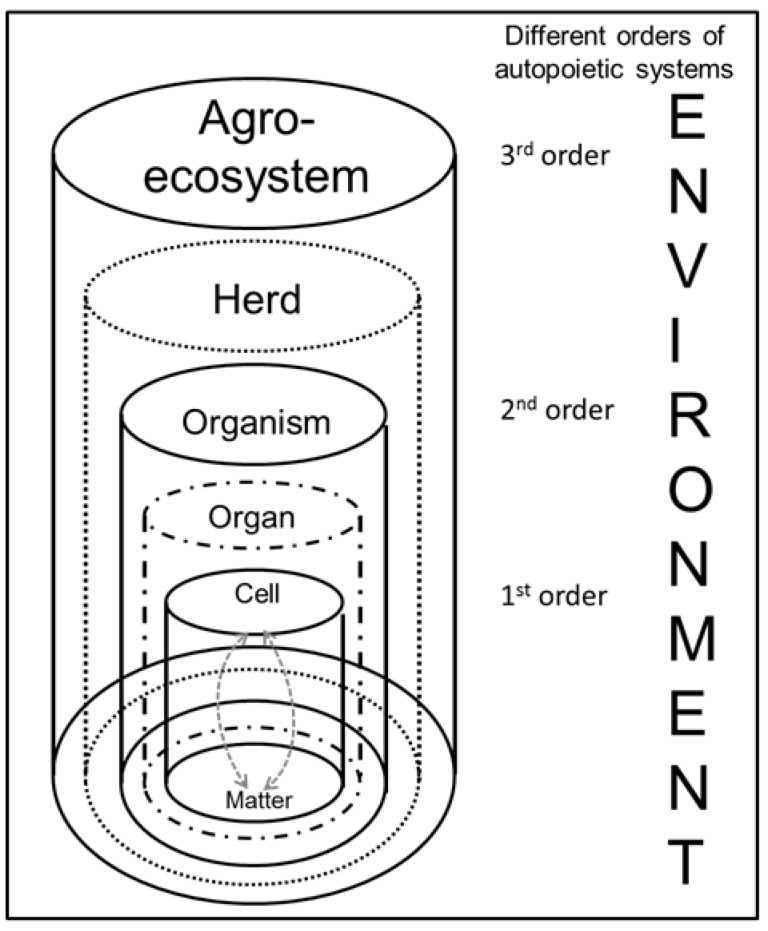
Hierarchical structure of autopoietic living systems of different orders within an agro-ecosystem.

Changes in the amounts and patterns of nutrients flowing through body compartments where these nutrients are degraded, synthesized, or stored, reflect the large day to day variation in nutrient availability the metabolism has to deal with. Metabolic disorders primarily develop when discrepancies exist between what is needed to sustain a certain balance and what is supplied from various sources, and when the organism cannot cope appropriately with these discrepancies. Discrepancies are not limited to single substrates but encompass a wide range of factors and processes, including interactions between different substrates within and between different compartments on different scales. The complex systems in living organisms operate both on a level of time and of space. The hierarchy of systems or scales is introduced because it corresponds to our subjective views of a system based usually on our various discrete experimental viewpoints. In their famous work, Oppenheim and Putnam [149] conceived of nature as being constituted by a hierarchy of objects that in turn, defined a hierarchy of distinct sciences. In agricultural and animal science, multiscale approaches inspired from theoretical physics have been developed in an essentially unidirectional (bottom-up) approach to integrate parameters at a given scale into reduced reproduction at higher scales [150]. However, lower-scale properties are also directly coupled with properties at a higher scale. The very complexity of living systems and biological functions lies partly in the presence of these bi-directional feedbacks between the upper and lower scale. An illustration of the hierarchical structure of living (autopoietic) systems is presented in Figure 1.

**Figure 2:**
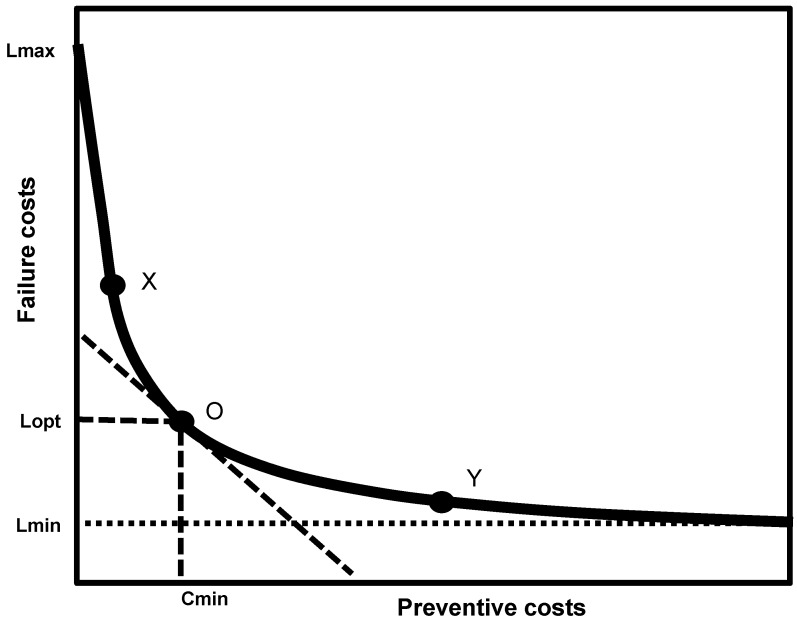
Schematic representation of the relation between failure and preventive costs [6].

Based on basic biological considerations by Maturana and Varela [1] concerning the characteristics of self-organizing autopoietic systems, reworked, and reformulated by Razeto-Barry [70], the vertical structure of living systems in agriculture can be also described as a hierarchy of scales. An example is provided in Figure 2 with the basic level of cells (1); interacting with material elements, and organizing themselves in tissues and organs (2) as superordinate systems, integrated in a whole organism (3); which is part of a herd (4); while the herd is an intermediary level between the single animal and the agro-eco-system (5). Microbiologists would probably see the genome as the basic level [150], while agriculturalists might extend the hierarchy of systems to far larger scales [151]. In the current context, it is primarily important to bear in mind that each superordinate system acts as the environment for the corresponding sub-system. It is the duality of the perspectives in particular which enables a more comprehensive understanding of biological processes. Without the whole living organism, its parts—the organs and the cells—could not exist or be sustained. Thus, the living system continuously produces the components, which constitute it by itself. Furthermore, these components steadily sustain and regenerate the superordinate system [152].

From a biological-systemic perspective, an organism is an integral and functional system in continual process of exchange with the environment. It represents an entity with a functional integrity [153]. Maintenance of the entity is an active process of self-organization which requires resources. In striving for maintenance, external material is regulated by the living systems following to their own principles and purposes, while integrating and transforming the material within the system. This principle is valid for the single cell as well as for the organism and for the farm as an agro-ecosystem. For instance, cells adapt their own metabolism and their functional capacity to the availability of external resources surrounding the cells [154]. To a certain degree, cells are able to perceive the availability of nutrients directly and transform this information into modified metabolic flux rates. Thus, processes are not exclusively determined by internal dispositions (e.g., genome or proteome) and conditions (e.g., anabolic or katabolic metabolism) or by impacts of the physical environment (e.g., availability of nutrients or external stressors). Living systems react due to the way the system is configured and adapt constantly to their environment. They select the appropriate components from the environment and give them a meaning of incentives, objects of perception, and interests [152].

Nutrients that would enable the individual animals to meet their requirements efficiently and comprehensively on a daily basis are neither available in the wild nor in livestock farming. Animals can rely on different metabolic pathways by which nutrient deficits or surplus can be compensated for an extended time period. Ability and skills to deal with nutrient imbalances are decisive if the animal is to succeed trying to adapt to inappropriate and varying supply conditions. Metabolic and reproductive disorders, diseases, and mortality indicate that the cows’ ability to adapt to variations in its environment is overstressed due to disturbances in the interdependency between super- and sub-ordinated systems and their dynamic transition processes. Disorders and diseases can be understood as negative bodily occurrences where a part of the organism fails to perform one of its biological functions appropriately. This interpretation corresponds with the concept of biological malfunction, recently described by Saborido and Moreno [17]. In this concept, biological functions are interpreted as specific causal effects of a part or a trait that contributes to a complex web of mutual interactions, which, in turn, maintain the organization and, consequently, the part itself.

Change and disturbance occur both between and on all scales. Due to a large variation in composition and ingredients of: feedstuffs, diets, daily feed intake, as well as digestive and absorbent capacities, a variable supply from the intermediate metabolism meets a variable need in nutrient and energy requirements in the various compartments of the body. The gap between demand and supply has to be covered by regulatory processes which for their part consist of influencing factors with respect to input, partitioning, and output variables. Dairy cows differ considerably in their adaptability to nutritional changes and metabolic disturbances, particularly to NEB. Last but not least, this is evident through large differences in prevalence and incidence of metabolic disorders despite similar environmental conditions. The reasons behind the differences in adaptability are manifold. How to evaluate the degree to which adaptation occurs in this specific situation, taking into consideration the large intra- and inter-individual variation in the nutrient flow that takes place on and between various scales, is therefore of particular interest. This is also true for various other factors involved in the adaptation process. The large individual differences in input, partitioning, and output variables indicate a variability of metabolic processes on various scales that cannot be narrowed to a single factor but which needs to encompass the interconnectedness of variables and the nutrient flow. Added to this is the fact that most problems which challenge organisms have a number of equivalent solutions, which can be thought of as existing in a vast neutral space [155]. A neutral space is a collection of equivalent solutions to the same biological problem, embodied in the biological systems ensuring an organism’s survival. This is the case for the multiple levels of biological organization. Understanding the structure of neutral spaces is critical to understanding the robustness of biological systems and to what extent the robustness itself can evolve.

A limited availability of nutrients and energy provokes severe competition between different tissues in the need for nutrients to sustain their various functions. Limitations require partitioning, and partitioning requires prioritization in guiding the nutrient flows, and ensuring that the demands of other cells, tissues, and organs within the organism are not completely neglected [30]. Thus, there is a need to prevent ruinous competition between sub-systems, to avoid being swamped by unwanted side reactions which clog the system or parasitic reactions by single organs to the expense of other organs which may cause the whole organism to collapse. Nutrient prioritization in early lactation to favor milk production over fertility is a reasonable strategy in biology [156]. As nutrition becomes scare, the lactating dam will preferentially invest the limited resources in the survival of living offspring rather than gambling on the oocyte that is yet to be ovulated, fertilized, and cared for during an entire gestation. Selection of high milk yields takes advantage of the genetically programmed readiness of the dairy cow to enter into a negative energy balance at the onset of lactation and to mobilize resources from its body tissues. However, selection has advanced into dimensions that are far beyond the initial intention to ensure nutrient supply to the off-spring via milk. Thus, there is no basic conflict between milk production to safeguard the off-spring on the one hand and self-preservation of the dam on the other. Life-threatening conflicts occur, when the gap between demand and supply gets to the stage where metabolic regulations are at risk of failing to mobilize the bodily resources needed to compensate for the deficits between nutrient output and intake. In case of failed regulation, the maternal ability to nourish the off-spring can be completely lost due to the death of the dam.

Adaptation depends *inter alia* on the degree to which the individual requirements of cows are met through the structure and organization of the farm system as the individual peculiarities of dairy cows mean that they differ considerably both in their general requirements and respecting their specific performance within the lactation course. To fulfil these demands, the farm system itself is in need of appropriate resources (high quality feed, investments, labor capacity, knowledge, *etc.*). What is needed on individual farms cannot be generalized but also depends on the specific farm system. At the same time, farmers faced with low milk prices are striving and fighting for the sustainability of their own farm system in a global market where all dairy farmers produce more or less the same commodity, thereby placing high pressure on production costs.

## 9. Challenged Farm Management

Intense selection for milk production has resulted in an immense priority for the high-producing dairy cow to partition energy to milk, at the cost of body reserves. This has resulted in an excessive negative energy balance (NEB) and high prevalence of metabolic disorders and poor reproductive performance. Thus, milk production, animal health, and reproductive performance in high-producing dairy cows generate conflicting interests. Traits in relation to cow health have only recently been included in the selection criteria on the basis that considerable genetic diversity with respect to health and reproductive performance exists in the current population [67]. However, functional traits have a comparably low heritability thus questioning whether such approaches will bring the desired improvements in the foreseeable future. In the meantime, animals will continue to suffer from production diseases while milk yields will further increase and linked with the negative side effects. The preparedness of the dairy cow to enter into a negative energy balance in early lactation while mobilizing nutrients from body tissues is an essential precondition for a high milk performance in the total lactation period. Apparently, dairy cows do not possess an effective feedback loop, e.g., to reduce milk yield and depletion of body reserves to a degree that prevents metabolic disorders and does not compromise the survival of the cow itself. Thus, it is the responsibility of the farm management with their various organizational skills and resources to avoid overstretching the gap between demand and supply. The crucial question is: when is the border which endangers the ability of individual animals to sustain themselves crossed? As this paper’s concept explains, such thresholds can neither be generalized nor predicted for the individual animal.

As milk yield increases, the management challenge of meeting a cow’s dietary nutrient needs becomes greater. This implies that increases in milk yield achieved through genetic selection requires improving the entire availability of nutritional resources. However, not all high yielding cows are capable of achieving an adequate energy intake, especially under grass-based management systems [157]. While energy requirements have increased more rapidly than dry matter intake (DMI), the nutrient density of diets has increased simultaneously, enhancing the risk of subacute ruminal acidosis [158]. Although advancements have been made in feeding practice to minimize the risk of metabolic diseases, the periparturient period continues to present some of the greatest challenges in the animals’ health [107]. Nutritional approaches vary enormously between farms and in many instances are failing to meet the more exacting demands on modern dairy cows [159]. Too many farmers are still concerned about feed costs per ton when the real focus should be on feed cost per liter milk produced. Larger herds usually result in more cows managed per available staff member. Declining availability of qualified dairy staff has added concerns that cows are receiving less individual attention, which is likely to have contributed to the high level of metabolic and fertility disorders. Dry cows are still often considered as second-class citizens or nonworking cows and thus receive little attention and poor-quality forage. Separation of dry cows into an independent group or into sub-groups is not readily practiced. Dry cows are often housed in a low-producing group [67].

Given that nearly all cows experience reduced feed intake and loss in body condition postpartum, there is a large variation between cows in the extent and duration of negative energy balance. Because the energy balance calculation requires knowledge of feed intake and digestibility, milk composition, and weight; in farm practice, energy balance values can often not be calculated due to lack of data on these components. Correspondingly, farm management is often eager to follow general recommendations instead of trying to adjust feeding regimes to the degree of NEB and the variation between cows, as this is easier. In the light of highly variable initial and boundary conditions in farm practice and limited availability of resources in dairy management, it is more than questionable whether “best practice” is still a realistic and valid concept. There is much evidence that specific measures operating under certain conditions are not necessarily effective, when compared with others in achieving the same purpose. Feeding higher-concentrate diets in close-up groups to meet increasing energy needs, is often practiced but recognized as decreasing the feed intake due to high availability of propionic acids which provokes satiety [73]. This situation can be counterproductive at a time when cows are accelerating their milk output, which accelerates lipid mobilization and results in associated negative consequences. On the other hand, excessive intakes of energy relative to requirements in late lactation and dry period is almost certainly expected to increase the risk of “fat cow” or “fatty liver” syndrome [77] and increase the risk of insulin resistance in cows and to elicit changes similar to obesity and type II diabetes [160]. Besides the availability of energy, the relevance of the dietary provision of metabolizable protein has been emphasized, based on strong indirect evidence backing the importance of the maintenance of maternal protein stores on long-term health, productivity, and reproduction [119].

With the continued challenges to cows navigating the transition period, some researchers have considered altering the dry period to minimize metabolic changes. Shortening or even skipping the dry period improves dry matter intake peripartum, reduces milk production in early lactation, improves energy balance, and reduces the number of days postpartum till resumption of ovarian activity [161,162]. A recent meta-analysis of 24 studies manipulating dry period length showed lower milk production, improved energy balance, and decreased risk of ketosis, but no difference in other diseases or fertility [163]. In a recent study by Chen *et al.* [164], cows with a 0-d dry period had an improved metabolic status in early lactation, indicated by lower plasma concentrations of non-esterified fatty acids, greater plasma concentrations of glucose, insulin-like growth factor-I, insulin and lower mRNA expression of pyruvate carboxylase in the liver, compared with cows with a 30-d or 60-d dry period. However, adoption of shorter dry periods has been limited because of the milk loss concerns.

A way to reduce the imbalance between nutrient supply and demand in early lactation is not only to improve the first but also to temporarily decrease the latter. While most dairy cows are milked twice daily, it is not uncommon in intensive dairying systems to increase milking frequency up to four times daily to increase milk production. Reducing milking frequency is much less common. However, the overall energy balance of cows during early lactation is improved with one-daily milking [165]. In dairying systems where an emphasis is placed on animal health rather than on milk production per cow, this practice might fit well under certain infrastructural farm conditions. Depending on the stage of lactation, breed, and parity the reduction in milk yield losses varies considerably and can amount to approximately 22% [166]. This figure has been revealed as an average milk loss across 30 different international short-term studies. Production expressed as milk solids (*i.e.*, combined fat and protein yields) is also lower with once-daily milking, despite the fact that milk fat and protein content of the milk increase [52,167]. The increase in fat and protein content is not sufficient to compensate for the decrease in milk volume. However, it may provide a tool to manage the metabolism and energy balance of cows during early lactation better [168], e.g., at highest negative energy balance period. Results from a study by Carbonneau *et al.* [169] revealed that blood concentrations of glucose were higher and concentrations of non-esterified fatty acids and ß-hydroxybutyrate were lower in partially milked cows compared to control cows during the treatment period. In other studies too, cows milked once a day showed an improvement in their metabolic profile [52] and immune function [59]. In contrast, cows subjected to an increased milking frequency were 1.4 times more likely to be classified as subclinically ketotic as the control cows [170].

One-sided selection for high milk yield is likely to produce cows that are increasingly more vulnerable to disease and poor fertility [171]. The authors concluded from different studies that negative genetic correlations between production and fitness traits increase in less favorable environments and that selection for increased yield has decreased the adaptability of modern cows. Keeping such animals in living conditions where farmers have increasingly less time available per cow demands even higher management skills. In situations of inferior management, unfavorable genetic correlations between milk yield and metabolic disorders are expected to be stronger than in herds with superior management. In a study allowing discrimination between the roles of genotype (G), environment (E) (e.g., feed caloric density and milking frequency), and GxE interactions, the effects of genetic merit and milking frequency were significant only in the groups that were fed rations with high caloric density [109]. However, signs of severe negative energy balance, poor protein balance, and low body condition scores were not concentrated in the highest producing cows. A lower feed caloric density and extra milking had strong unfavorable effects on both energy and protein balance, and emphasize the possible effect of mismanagement on animal health risks. Increased milking frequency increased milk yield, although only significantly when the cows received high caloric density rations. Milking the cows three times a day instead of twice significantly decreased energy balance. Thus, environmental factors like feeding management and milking frequency strongly influence the cows’ energy balances. High genetic merit cows seem more prone to allocate resources toward high fat output in times of nutrient-deficient diets and toward high protein output with high nutrient diets. High genetic merit for milk yield seems intrinsically connected with the allocation of resources from maintenance toward milk. To prevent compromise of animal health status; demands on the provision of appropriate feed and feeding conditions; farmers’ time and management skills are clearly increasing. In a direct comparison of low- and high-yielding genetic lines of cattle, at relatively similar levels of dry matter intake, there is no evidence of a difference in the fractional recovery of DE from intake energy or ME from DE [172]. Similarly, no differences were found in the efficiency of ME use for milk energy. Thus, increase in productivity due to higher milk performance per cow is based primarily on the proportional reduction in nutrient resources needed to cover the demands for maintenance. In terms of maintenance requirement, the high yielding cow is more efficient as maintenance cost per unit milk is diluted [173].

Against an industry-held view that milk output is the single most important aspect in an efficient dairy business, it is contended that producers need to refocus on factors which also affect profitability, mainly: nutrient efficiency, cow health, fertility, and longevity [6,174,175]. In animal nutrition, the most efficient use of nutrient resources is generally obtained when each animal is supplied with energy and nutrients according to their individual demands [40]. Both exceeding supply before calving and under-nutrition postpartum not only reduces efficiency but requires additional resources of regulation to maintain the internal processes within a certain range of homeostatic conditions [9,176]. Feeding farm animals according to their current requirements regarding nutrients and energy provides the best orientation for the farmer to gain a high efficiency in the use of nutrients while simultaneously reducing the risks of metabolic disturbances. However, to meet such a goal requires additional resources, amongst others: high quality feed, good size and type of stalls, suitable amount of eating space available to the cows or ventilation systems in place, and last but not least knowledge and skills to reduce possible gaps and compensate for deficits [12]. Thus market conditions which do not provide enough income for farmers and do not fully cover the expenditure during the production process threaten not only the sustainability of the farm systems but also that of the dairy cows and their functional integrity of organs and tissues through downward causation.

While farmers have to consider changes in the availability of resources, they can no longer ignore consumer concerns about animal health and welfare, and food safety. Increasing disadvantages of the intensification process and conflicts with the interests of other stakeholders call for a critical assessment of the sustainability of dairy production [177]. What is sustainable for agricultural systems is subject not only to system-inherent and self-referential demands but also to changes in the market. Thus, farm managers are not only agents of control but components of the agricultural system itself. The fact that market prices and consumer preferences are primarily dominated by forces beyond the farmers’ control makes the issue even more complex and somewhat confusing. In providing the essentials for remaining both competitive and sustainable, the farm management is restricted to only a few options; either to further reduce production costs per product unit or to obtain a higher selling price. As long as qualitative traits such as the production of “healthy” food from healthy cows are not honored by premium prices, farm management has to focus on how to improve the overall efficiency of production processes [178]. This cannot be achieved solely by addressing the milk yield of dairy cows. In fact, farmers have to take into account the cost-benefit relationships on various scales, including failure costs caused by metabolic disorders and production diseases as well as the preventive costs needed to reduce these. Expenditures for preventive measures can be seen as additional input to reduce monetary losses of disorders and diseases [6]. The higher the preventive costs, the lower the failure costs and *vice versa* (see Figure 2). If no preventive and control measures are taken, the losses due to disease are at their maximum (Lmax). With maximum prevention, the failure costs due to disease will be at a minimal (Lmin). Because the relationships between prevention and failure is not linear, there is an optimal level of control (Lopt). To gain profit from reduced prevalence of production diseases, the farm management has to strive for an optimum between failure and preventive costs. Due to the various and highly varying factors involved in the development of productivity and in the development of disorders, and due to the large differences in the availability of resources mentioned above, such a balance is different for each farm system. Furthermore, the balance varies in relation to changes in the environment of the farms, especially regarding changes in food and feed markets.

It is in the nature of much-circulated general recommendations that they only marginally address the context in which the biological processes take place. Correspondingly, they may appear counterproductive, especially when they distract the farm management from looking for farm specific solutions. The latter should rely on valid data which unfortunately are often not available to the desired quality, probably because data acquisition itself is very time consuming and reflecting upon data needs much mental energy [179]. Moreover, changes in perspectives are required to implement and assess bilateral adaptation processes, on the one hand by the animals towards their living conditions and on the other by the farm management in relation to the specific demands and limitations in the adaptation capacities of dairy cows, to the benefit of both animals and farmers.

## 10. Challenged Agricultural Science

Agricultural research and animal science as a sub-discipline deal with agricultural systems and with socio-technical and socio-economic circumstances as well as the superordinate systems which influence them. Criteria for assessing the quality of agricultural research are shaped on a problem-oriented systems research basis [180]. This consists of the close connection between basic and applied research, the multi- and interdisciplinary character of agricultural research and the orientation of research towards the solving of issues relevant to society. Regarding the high prevalences of metabolic disorders and associated production diseases in dairy production, agricultural science so far cannot claim to have been able to reduce health problems. Despite tremendous scientific efforts in the past, the situation has not improved adequately. Animal scientists might at least claim that without technical and scientific progress in gaining more insights and knowledge about the pathophysiological processes, the situation might have become even worse. However, lack of success in reducing production diseases does not meet goals set by agricultural scientists and there is thus an impetus to reflect on whether the previous approaches in dealing with health problems are still appropriate to the questions on hand. In the first instance, a reductionist approach has been employed, accumulating large quantities of detailed information with little attempt to incorporate it into a broad view of the whole subject. Thus, scientific efforts have provided a wealth of knowledge but to a large extend, this information has not been integrated into coherent biological models. Members of animal science in particular have not done well to integrate output productivity and negative side effects with respect to animal welfare [181].

In the case of adaptation to metabolic challenges too, there is a predominant tendency to reduce the differences between animals’ ability to cope with nutritional and metabolic challenges to single factors, with a priority to differences in the genome (e.g., [47,182,183]). Based on the methodological advances made in the human genome project, and the need to interpret findings in a more biologically meaningful fashion, “system biology” has emerged as a new approach [184]. In the meantime this has been applied to many species, including dairy cows. The pace at which transcriptomics has expanded has forced the discipline of genomics to embrace the notion of holism as far as animal function is concerned [185]. The new approach focusses on the gene/protein expression (proteomics) on the cell/tissue level and the associated changes in biological functions. It tries to combine omics-generated data with mathematics and computer science to gain knowledge of biological behavior of a cell in either a bottom-up or top-down direction [186]. Recent developments in technology have paved the way for new modelling approaches at various levels of complexity [115,187,188]. One of the main objectives is to be able to predict the ability of an animal to respond to the nutritional limitations that arise from the environment in which it is placed. The perspective is that of incorporating increasingly detailed descriptions of genotype via genomics, and of the downstream metabolic machinery via gene expression, proteomic and metabolomic information [115]. As indicated by the authors, this remains a daunting task; not only due to serious methodological hurdles. The authors also acknowledge the danger that even if all the omic information were available, it might be still too complex for the minds of scientists. Whilst generating biological information about dairy cattle through the application of genome-enabled tools is no longer a bottleneck, the biggest challenge is to interpret findings in a more biologically meaningful fashion and eventually to communicate biological knowledge systematically by linking the genome to the whole organism [188]. The authors admit that the use of the omics’ tools alone cannot provide a full picture of the biological system and can thus be misleading when interpreting biological outcomes, while scientists are still struggling to interpret the results from any one of the omics’ technologies in a holistic and unbiased way.

While many genome-wide association studies have frequently shown that they do not explain the majority of the variation in whole-animal phenotypes, it has become clear that the relationship between the genome and the phenome is best characterized in terms of causal interdependency [150]. Genes are often found to possess multiple functions, which are sometimes critically dependent on the context and the specificity of enhancers for promotor sequences is often surprisingly loose [189]. Thus, even for fixed genotypes and environments, a large variability of phenotypes can occur across a population [190]. According to McNamara [181], “systems biology” means different things to different people. At minimum, it is the recognition that each piece of the system has a specific function related to the outcome of the entire system, not just the subsystem in which the molecule acts. For Cornish-Bowden *et al.* [146], the term “system biology” is little more than a euphemism for gathering even more details on an even larger scale, and not, as it should be, the study of biological systems as systems rather than as a collection of components. The more complex the level at which one seeks to explain a living system, the greater the need to examine the network of interactions.

In opposition to the omics’ approach, it might be further remarked that in search for general principles and generalizable regularities, the mathematical approach is faced with the tremendous variation that occurs on the nutrient pathways from nutrient intake to utilization and secretion via milk. The variation is increased further by the interconnectedness of the variables on different scales, organized by various forward and feedback loops. As origin, scales of time and space, control, and functional significance of fluctuations in biological systems are largely unknown, it is questionable whether the genomic approach will be able to achieve biologically meaningful aggregated phenotypic descriptions in a foreseeable time period, let alone provide a valid base for decision-making processes on a farm level.

It has become increasingly clear that understanding nutrient partitioning is central in grasping the complexity of adaptation to shortages and imbalances in nutrient supply in relation to requirements, and thus of the ability of dairy cows to cope, especially when challenged with abrupt and unforeseeable changes. This refers not only to the nutrient partitioning within the animals but also to the level of the farm. In looking for approaches to gain knowledge in predicting nutrient partitioning in dairy cows, animal scientists often recognize the central role of the genotype. This however, leads to the question, how a valid prediction regarding the reactions of animals with a high variability in their medical history towards more or less unpredictable challenges can be obtained. Although the degree of deficits might be comparable between cows, for instance in the extent of NEB, there are large differences in the possible reasons behind the deficits and thus in the reactions and adaptation processes of the individual animals as outlined above. In the light of metabolic disorders and production diseases, affecting more than every other cow in early lactation to a varying degree, expectations concerning success in gaining predictions on the capacities to adapt are—to formulate it cautiously—astonishing, at least from the perspective of animal nutrition and animal health. Probabilities for successful adaptation can be at best predicted on the population level. This however, does not necessarily mean that what might be expected on the population level is valid for the corresponding farm level where adaption takes place in reality.

To look beyond narrowly molecular perspectives for answers to biological questions, it is important for metabolic adaptation not just to look at the total sum of the individual parts, but how they interact, and especially if they interact successfully in preventing the animals from suffering metabolic disorders and associated production diseases. A widespread view is that causation operates in an exclusively bottom-up way, from the microscopic to the macroscopic. Adherents of such a view often express perplexity at the idea that causation might even run in the reverse direction [150]. In tracking the flows of causal influence that might overstress the ability of an animal to adapt and increase the risk of metabolic disorders, it should be evident that the main causalities do not occur on the cell or genome level and thus cannot be solved there. In the first place, difficulties to adapt are due to extended and dynamic gaps between nutrient requirements and nutrient supply that already begin with the nutrient partitioning on the farm or even a larger scale. The central role of the whole organism, the genome being only a minor part, is to deal with the challenges deriving from the gaps between synchronized and tight causal coupling among the subsystems on the different scales. Correspondingly, it is the organism as a whole that succeeds or fails in adapting to nutritional change and disturbance.

Biological systems as a whole embody solutions to important biological challenges. Complex systems conveniently provide a conceptual framework and effective tools to solve problems and to decouple emergent and immergent features from molecules to organisms and back. The latter is also described as downward causation meaning the necessity for all processes at the lower level of hierarchy to act in conformity to the laws of the higher level. This means that some macro-level constraints are expected to cascade back onto micro-levels, the macro-level being itself an emergence [191]. Accordingly, pathological symptoms indicating disturbed adaptation to nutritional and metabolic changes cannot be reduced to a single factor but result from the complex interactions between various processes at different scales, thus expressing the present pathophysiology state of the whole organism.

Biological systems are goal-driven and above all they strive to survive and sustain. Facing a restricted availability of resources, while being confronted with extended change and disturbance, some animals are more successful than others at coping. However, animals that show competitive advantages in a specific unbalanced situation do not necessarily have an advantage in a quite different nutritional situation and might not show better results in the long run with respect to lifetime efficiency. The fact that metabolic disorders and production diseases have been occurring for a long time might suggest looking for solutions in the long run, e.g., breeding measures. However, metabolic disorders and production diseases are an instant problem, causing suffering, and thus require shot-term reactions and counteractive measures. Metabolic problems that derive from inappropriate and unbalanced nutrient supplies cannot be solved by selections between different genotypes but can only be reduced by the farm management in the specific context and environmental conditions. There is a significant body of knowledge on how to provide optimal farm management and prevention of bovine metabolic disorders and production diseases. For instance, tremendous efforts have been made in animal nutrition to assess and predict nutrient requirements of cows in their various life stages and following different animal and environment related variables. However, knowledge on how to adapt requirements to each unique case does not provide information on how to deal with the intra- and inter-individual variability in both nutrient demands and nutrient supply within a herd, and how to support endangered animals in coping when facing a large gap between demand and supply.

In the light of high prevalences and incidences of metabolic disorders and production diseases, it is obvious that many farm systems do not provide adequate solutions to the apparent problems; they thus overstretch the adaptation capacities of their animals. Farm systems themselves are facing fierce competition and have to adapt to change and disturbance; amongst others in feed and food markets. Farmers thus often lack the resources in terms of quality feed, labor time, investments, know how, *etc.*, needed to improve the status of animal health and welfare. Partitioning of the available resources in the most efficient way is not only a challenge for the animal but also for farm management. It can be expected that farmers would be willing to invest more in animal health and welfare if they could expect a competitive advantage. A large variation between farms regarding failure and preventive costs indicate that there is room for improvement on many farms meaning an appropriate balance which benefits a farm’s profitability.

The task of agricultural and animal science should be to identify the most effective preventive measures and/or therapeutic options for farm animals but also the most cost-effective and economic approach in the corresponding complex farm system. In the first place, trade-offs have to be addressed on the scale where they emerge (principle of subsidiarity), following a bi-directional approach which simultaneously reflects upward and downward causation. Applied agricultural and animal science should support the farmer in striving for the goal to find an economic balance between productivity and animal health and welfare to the benefit of the animals, the farmers, and the prosperity of the common good. This can only be obtained by the development of more comprehensive concepts and evidence based solutions which simultaneously consider a larger number of causal relationships to discover regularities beyond the scope of individual scientific disciplines.

## 11. Conclusions

The pathophysiology of metabolic disorders and postpartum diseases is quite complex, interrelated with many processes within or outside the intermediary metabolism, they are also multifactorial and often driven by several interconnected risk factors. In this way, it is difficult for both scientists and the farm management to grasp the complexity and make the appropriate decisions to improve the adaptation capacity of dairy cows, particularly in the transition period. Metabolic processes in early lactation are dominated by a pull effect originating from the high glucose demand of the epithelial cells in the udder and by a large variation on the various process levels. The capacity of the dairy cows to cope with unbalanced situations between requirements and supply is limited and often exhausted. Especially in the transition period, dairy cows face varying initial conditions (e.g., body condition, medical history) and different boundary conditions (e.g., feeding and housing conditions) which interact with physiological regularities and thus result in highly variable outcomes. As a result, the interconnectedness of numerous factors on different scales create a large variation in adaptation capacities and in the occurrence of metabolic disorders and production diseases both on the animal and dairy farm levels.

It is concluded that the fundamental principles of metabolism are based on network properties which are able to contribute to the adaptability of the organism and not on the properties of single components. A metabolic network is a functional union of the different organs and tissues involved, providing a responsiveness to metabolic change and disturbance as a functional unit or as a whole organism, respectively. An understanding of symbiosis implies that the inter-tissue competition becomes an insufficient explanation for metabolic function but must incorporate a wide range of very intimate modes of cooperation. In a new perspective, the object of biological investigation becomes the process that pervades multiple levels of organization. Cells, and tissues must be understood as sub-systems within the framework of organs, and these sub-systems are embedded within broader organismal systems of interconnected and communicating structures. This represents a cooperative aspect of life and carries much importance in relation to fitness and reproduction.

The ability to cope with suboptimal living conditions and a large gap between nutrient supply and demand cannot be left to the dairy cow itself. It lies in the first place under the responsibility of the farm management to regulate nutrient and energy input as well as output to prevent the exhaustion of this adaptation capacity. On the other hand, dairy farming is a business that people engage in for financial gains. Dairy production has capitalized on the metabolic drive of the dairy cow to ensure provision of milk to the calf by selecting for increasingly higher milk production. This strategy has been very successful. Unfortunately, the goals of economic efficiency and optimal dairy cow health are often in conflict. Metabolic disorders in early lactation indicate an overstressed ability of the animals to adapt to living conditions that do not appropriately provide the specific nutrient and energy requirements. Dairy cows will succeed more easily in adaptation and in avoiding dysfunctional processes in the transition period when the gap between nutrient and energy demands and supply is restricted. The fact that on average, one in two cows succumbs to health problems during the transition period highlights the fragility of many production systems. From these previous considerations, it seems obvious that animals within a herd will be better able to adapt in order to survive, if appropriate resources and living conditions are offered which meet the individual requirements at the different stages to a high degree and if nutritional and other disturbances are reduced to a minimum. Any attempts to reduce prevalences of metabolic disorders and associated production diseases should rely on a continuous and comprehensive monitoring of appropriate indicators on the farm level. If low prevalences of metabolic disorders and production diseases would go along with a competitive advantage, it would support the farm management in providing appropriate living conditions. As a first step, low prevalences for the most relevant production disease should be established as a separate production goal, continuously assessed by appropriate criteria and monitored as an emergent health output of the farm practice. Furthermore, those farms able to combine a low prevalence of production diseases with a high productive output should be rewarded by premium prices. In the light of a lack of success in a strong reduction in the prevalence of metabolic disorders and production diseases; the deficiencies in reductionist approaches on the farm level are becoming increasingly apparent, challenging the disciplines of agricultural and animal science. Thus, scientists should ask themselves whether the focus on single aspects has contributed to promoting the tendency of oversimplification and of widely ignoring the context in which adaptation processes take place. One of the central questions should be to what degree each of the subsystems contributes to the benefit of the superordinate systems and *vice versa*.

## References

[1] Maturana H.R., Varela F.J. (1980). Autopoiesis and Cognition: The Realization of the Living.

[2] Piaget J. (1971). Biology and Knowledge: An Essay on the Relations between Organic Regulations and Cognitive Processes.

[3] Broom D.M. (1993). Assessing the welfare of modified or treated animals. Livest. Prod. Sci..

[4] Wingfield J.C., Romero L.M., McEwen B.S. (2000). Adrenocortical responses to stress and their modulation in free-living vertebrates. Handbook of Physiology, Section 7, Coping with the Environment: Neural and Endocrine Mechanisms.

[5] Leroy J.L., Vanholder T., van Knegsel A.T.M., Garcia-Ispierto I., Bols P.E.J. (2008). Nutrient prioritization in dairy cows early postpartum: Mismatch between metabolism and fertility?. Reprod. Domest. Anim..

[6] Hogeveen H. Costs of production diseases. Proceedings of the 27th World Buiatrics Congress.

[7] Drackley J.K. (1999). Biology of dairy cows during the transition period: The final frontier?. J. Dairy Sci..

[8] Bell A.W. (1995). Regulation of organic nutrient metabolism during transition from late pregnancy to early lactation. J. Anim. Sci..

[9] Sordillo L.M., Raphael W. (2013). Significance of metabolic stress, lipid mobilization, and inflammation on transition cow disorders. Vet. Clin. North Am. Food Anim. Pract..

[10] Ametaj B.N. (2014). Metabolic disorders of dairy cattle. Encyclopedia of Life Support Systems (EOLSS): Veterinary Science.

[11] Mulligan F.J., Doherty M.L. (2008). Production diseases of the transition cow. Vet. J..

[12] Oetzel G.R. (2014). Undertaking nutritional diagnostic investigations. Vet. Clin. North Am. Food Anim. Pract..

[13] Herdt T.H. (2013). Metabolic diseases of dairy cattle. Vet. Clin. North Am. Food Anim. Pract..

[14] Brand F.S., Jax K. (2007). Focusing the meaning(s) of resilience: Resilience as a descriptive concept and a boundary object. Ecol. Soc..

[15] Darnhofer I. (2010). Strategies of family farms to strengthen their resilience. Environ. Policy Gov..

[16] Di Paolo E. (2005). Autopoiesis, adaptivity, teleology, agency. Phenomenol. Cogn. Sci..

[17] Saborido C., Moreno A. (2015). Biological pathology from an organizational perspective. Theor. Med. Bioeth..

[18] Wingfield J.C., Schulkin J. (2004). Allostatic load and life cycles: Implications for neuroendocrine control mechanisms. Allostasis, Homeostasis, and the Costs of Physiological Adaptation.

[19] Montévil M., Mossio M. (2015). Biological organisation as closure of constraints. J. Theor. Biol..

[20] Bernard C. (1865). Introduction à L’étude de la Médecine Expérimentale.

[21] Bernard C. (1878). Leçons sur les Phénomènes de la vie Communs aux Animaux et aux Végétaux.

[22] Cannon W.B. (1929). Organization for physiological homeostasis. Physiol. Rev..

[23] Ashby W.R. (1956). An Introduction to Cybernetics.

[24] Wiener N. (1948). Cybernetics: Or Control and Communication in the Animal and the Machine.

[25] Cannon W.B. (1932). The Wisdom of the Body.

[26] Selye H. (1973). Homeostasis and heterostasis. Perspect. Biol. Med..

[27] Waddington C.H., Waddington C.H. (1968). The basic ideas of biology. Towards a Theoretical Biology: Prolegomena.

[28] Letelier J.-C., Cárdenas M.L., Cornish-Bowden A. (2011). From l’homme machine to metabolic closure: Steps towards understanding life. J. Theor. Biol..

[29] Varela F.G., Maturana H.R., Uribe R. (1974). Autopoiesis: The organization of living systems, its characterization and a model. Biosystems.

[30] Bauman D.E., Currie B.W. (1980). Partitioning of nutrients during pregnancy and lactation: A review of mechanisms involving homeostasis and homeorhesis. J. Dairy Sci..

[31] Mrosovsky N. (1990). Rheostasis: The Physiology of Change.

[32] Knight C.H., Beever D.E., Sorensen A. (1999). Metabolic loads to be expected from different genotypes under different systems: Metabolic stress in dairy cows. Br. Soc. Anim. Sci. Occas. Publ..

[33] Sterling P., Eyer J., Fisher S., Reason J. (1988). Allostasis: A new paradigm to explain arousal pathology. Handbook of Life Stress, Cognition and Health.

[34] McEwen B.S. (1998). Stress, adaptation, and disease: Allostasis and allostatic load. Ann. N. Y. Acad. Sci..

[35] McEwen B.S., Wingfield J.C. (2003). The concept of allostasis in biology and biomedicine. Horm. Behav..

[36] Herdt T.H., Joshi N.P., Herdt T.H. (2006). The history and influence of the ICPD. Production Diseases in Farm Animals: 12th International Conference.

[37] Knaus W. (2009). Dairy cows trapped between performance demands and adaptability. J. Sci. Food Agric..

[38] Rauw W., Kanis E., Noordhuizen-Stassen E., Grommers F. (1998). Undesirable side effects of selection for high production efficiency in farm animals: A review. Livest. Prod. Sci..

[39] LeBlanc S., Dalin G. (2013). Managing critical periods—transition dairy cows. Book of Abstracts, 15th Conference on Production Diseases in Farm Animals.

[40] Van Saun R.J., Sniffen C.J. (2014). Transition cow nutrition and feeding management for disease prevention. Vet. Clin. North Am. Food Anim. Pract..

[41] Dohoo I.R., Wayne Martin S. (1984). Disease, production and culling in Holstein-Friesian cows: IV. Effects of disease on production. Prev. Vet. Med..

[42] Andersson L., Emanuelson U. (1985). An epidemiological study of hyperketonaemia in Swedish dairy cows; Determinants and the relation to fertility. Prev. Vet. Med..

[43] Erb H.N. (1987). Interrelationships among production and clinical disease in dairy cattle: A review. Can. Vet. J..

[44] Melendez P., Marin M.P., Robles J., Rios C., Duchens M., Archbald L. (2009). Relationship between serum nonesterified fatty acids at calving and the incidence of periparturient diseases in Holstein dairy cows. Theriogenology.

[45] Ospina P.A., Nydam D.V., Stokol T., Overton T.R. (2010). Evaluation of nonesterified fatty acids and beta-hydroxybutyrate in transition dairy cattle in the northeastern United States: Critical thresholds for prediction of clinical diseases. J. Dairy Sci..

[46] Goff J.P., Horst R.L. (1997). Physiological changes at parturition and their relationship to metabolic disorders. J. Dairy Sci..

[47] Drackley J.K., Dann H.M., Douglas G.N., Guretzky N.A.J., Litherland N.B., Underwood J.P., Loor J.J. (2005). Physiological and pathological adaptations in dairy cows that may increase susceptibility to periparturient diseases and disorders. Italian J. Animal Sci..

[48] Van der Drift S.G.A., Jorritsma R., Schonewille J.T., Knijn H.M., Stegeman J.A. (2012). Routine detection of hyperketonemia in dairy cows using Fourier transform infrared spectroscopy analysis of β-hydroxybutyrate and acetone in milk in combination with test-day information. J. Dairy Sci..

[49] Kessel S., Stroehl M., Meyer H.H.D., Hiss S., Sauerwein H., Schwarz F.J., Bruckmaier R.M. (2008). Individual variability in physiological adaptation to metabolic stress during early lactation in dairy cows kept under equal conditions. J. Anim. Sci..

[50] Aitken S., Corl C., Sordillo L. (2011). Immunopathology of mastitis: Insights into disease recognition and resolution: Journal of Mammary Gland Biology and Neoplasia. J. Mammary Gland Biol. Neoplasia.

[51] Esposito G., Irons P.C., Webb E.C., Chapwanya A. (2014). Interactions between negative energy balance, metabolic diseases, uterine health and immune response in transition dairy cows. Anim. Reprod. Sci..

[52] Loiselle M.C., Ster C., Talbot B.G., Zhao X., Wagner G.F., Boisclair Y.R., Lacasse P. (2009). Impact of postpartum milking frequency on the immune system and the blood metabolite concentration of dairy cows. J. Dairy Sci..

[53] Wathes D.C., Cheng Z., Chowdhury W., Fenwick M.A., Fitzpatrick R., Morris D.G., Patton J., Murphy J.J. (2009). Negative energy balance alters global gene expression and immune responses in the uterus of postpartum dairy cows. Physiol. Genomics.

[54] Moyes K.M., Drackley J.K., Morin D.E., Rodriguez-Zas S.L., Everts R.E., Lewin H.A., Loor J.J. (2010). Mammary gene expression profiles during an intramammary challenge reveal potential mechanisms linking negative energy balance with impaired immune response. Physiol. Genomics.

[55] Burvenich C., Bannerman D.D., Lippolis J.D., Peelman L., Nonnecke B.J., Kehrli M.E., Paape M.J. (2007). Cumulative physiological events influence the inflammatory response of the bovine udder to *Escherichia coli* infections during the transition period. J. Dairy Sci..

[56] LeBlanc S.J., Osawa T., Dubuc J. (2011). Reproductive tract defense and disease in postpartum dairy cows. Theriogenology.

[57] Sheldon I.M., Cronin J., Goetze L., Donofrio G., Schuberth H.-J. (2009). Defining postpartum uterine disease and the mechanisms of infection and immunity in the female reproductive tract in cattle. Biol. Reprod..

[58] Huzzey J.M., Veira D.M., Weary D.M., von Keyserlingk M.A.G. (2007). Prepartum behavior and dry matter intake identify dairy cows at risk for metritis. J. Dairy Sci..

[59] Ster C., Loiselle M.-C., Lacasse P. (2012). Effect of postcalving serum nonesterified fatty acids concentration on the functionality of bovine immune cells. J. Dairy Sci..

[60] Hammon D.S., Evjen I.M., Dhiman T.R., Goff J.P., Walters J.L. (2006). Neutrophil function and energy status in Holstein cows with uterine health disorders. Vet. Immunol. Immunopathol..

[61] Sordillo L.M., Contreras G.A., Aitken S.L. (2009). Metabolic factors affecting the inflammatory response of periparturient dairy cows. Anim. Health Res. Rev..

[62] Sordillo L.M., Mavangira V. (2014). The nexus between nutrient metabolism, oxidative stress and inflammation in transition cows. Anim. Prod. Sci..

[63] Farney J.K., Mamedova L.K., Coetzee J.F., KuKanich B., Sordillo L.M., Stoakes S.K., Minton J.E., Hollis L.C., Bradford B.J. (2013). Anti-inflammatory salicylate treatment alters the metabolic adaptations to lactation in dairy cattle. Am. J. Physiol. Regul. Integr. Comp. Physiol..

[64] Huzzey J.M., Mann S., Nydam D.V., Grant R.J., Overton T.R. (2015). Associations of peripartum markers of stress and inflammation with milk yield and reproductive performance in Holstein dairy cows. Prev. Vet. Med..

[65] Abuelo A., Hernández J., Benedito J.L., Castillo C. (2015). The importance of the oxidative status of dairy cattle in the periparturient period: Revisiting antioxidant supplementation. J. Anim. Physiol. Anim. Nutr. (Berl.).

[66] Taylor V., Beever D., Wathes D., Kebreab E., Mills J. (2004). Physiological adaptions to milk production that affect the fertility of high yielding dairy cows. Dairying: Using Science to Meet Consumers’ Needs.

[67] Beever D.E. (2006). The impact of controlled nutrition during the dry period on dairy cow health, fertility and performance. Anim. Reprod. Sci..

[68] Robinson J.J., Ashworth C.J., Rooke J.A., Mitchell L.M., McEvoy T.G. (2006). Nutrition and fertility in ruminant livestock. Anim. Feed Sci. Technol..

[69] Collard B.L., Boettcher P.J., Dekkers J., Petitclerc D., Schaeffer L.R. (2000). Relationships between energy balance and health traits of dairy cattle in early lactation. J. Dairy Sci..

[70] Razeto-Barry P. (2012). Autopoiesis 40 years later. A review and a reformulation. Orig. Life Evol. Biosph..

[71] Grant R.J., Albright J.L. (1995). Feeding behavior and management factors during the transition period in dairy cattle. J. Anim. Sci..

[72] Tylutki T.P., Fox D.G., Durbal V.M., Tedeschi L.O., Russell J.B., van Amburgh M.E., Overton T.R., Chase L.E., Pell A.N. (2008). Cornell Net Carbohydrate and Protein System: A model for precision feeding of dairy cattle. Anim. Feed Sci. Technol..

[73] Allen M.S., Piantoni P. (2013). Metabolic control of feed intake: Implications for metabolic disease of fresh cows. Vet. Clin. North Am. Food Anim. Pract..

[74] Allen M.S. (2000). Effects of diet on short-term regulation of feed intake by lactating dairy cattle. J. Dairy Sci..

[75] Allen M.S., Bradford B.J., Oba M. (2009). The hepatic oxidation theory of the control of feed intake and its application to ruminants. J. Anim. Sci..

[76] Meyer U., Horstmann K., Kaske M., Flachowsky G. (2007). Inter- and Intra-Individual Variation of Feed Intake and Metabolic Parameters of Dairy Cows Related to Energy Supply.

[77] Dann H.M. (2004). Dietary Energy Restriction during Late Gestation in Multiparous Holstein Cows. Ph.D. Thesis.

[78] Lean I.J., Golder H.M., Hall M.B. (2014). Feeding, evaluating, and controlling rumen function. Vet. Clin. North Am. Food Anim. Pract..

[79] Proudfoot K.L., Veira D.M., Weary D.M., von Keyserlingk M.A.G. (2009). Competition at the feed bunk changes the feeding, standing, and social behavior of transition dairy cows. J. Dairy Sci..

[80] Cook N.B., Bennett T.B., Nordlund K.V. (2004). Effect of free stall surface on daily activity patterns in dairy cows with relevance to lameness prevalence. J. Dairy Sci..

[81] Kuhla B. (2012). Central Regulation of Feed Intake in Early Lactation.

[82] Merchen N.R., Elizalde J.C., Drackley J.K. (1997). Current perspective on assessing site of digestion in ruminants. J. Anim. Sci..

[83] Kamra D.N. (2005). Rumen microbial ecosystem. Curr. Sci..

[84] Tajima K., Aminov R.I., Nagamine T., Matsui H., Nakamura M., Benno Y. (2001). Diet-dependent shifts in the bacterial population of the rumen revealed with real-time PCR. Appl. Environ. Microbiol..

[85] Brown M.S., Krehbiel C.R., Galyean M.L., Remmenga M.D., Peters J.P., Hibbard B., Robinson J., Moseley W.M. (2000). Evaluation of models of acute and subacute acidosis on dry matter intake, ruminal fermentation, blood chemistry, and endocrine profiles of beef steers. J. Anim. Sci..

[86] Pers-Kamczyc E., Zmora P., Cieślak A., Szumacher-Strabel M. (2011). Development of nucleic acid based techniques and possibilities of their application to rumen microbial ecology research. J. Anim. Feed Sci..

[87] Stewart C.S., Flint H.J., Bryant M.P., Hobson P.N., Stewart C.S. (1997). The rumen bacteria. The Rumen Microbial Ecosystem.

[88] Dijkstra J., Kebreab E., Mills J.A.N., Pellikaan W.F., López S., Bannink A., France J. (2007). Predicting the profile of nutrients available for absorption: From nutrient requirement to animal response and environmental impact. Animal.

[89] Nozière P., Hoch T., Kebreab E., Nozière P., Hoch T. (2006). Modelling fluxes of volatile fatty acids from rumen to portal blood. Nutrient Digestion and Utilization in Farm Animals: Modelling Approaches.

[90] Dijkstra J., Boer H., van Bruchem J., Bruining M., Tamminga S. (1993). Absorption of volatile fatty acids from the rumen of lactating dairy cows as influenced by volatile fatty acid concentration, pH and rumen liquid volume. Br. J. Nutr..

[91] Rabelo E., Rezende R.L., Bertics S.J., Grummer R.R. (2003). Effects of transition diets varying in dietary energy density on lactation performance and ruminal parameters of dairy cows. J. Dairy Sci..

[92] Dijkstra J., Ellis J.L., Kebreab E., Strathe A.B., López S., France J., Bannink A. (2012). Ruminal pH regulation and nutritional consequences of low pH. Anim. Feed Sci. Technol..

[93] Stein S.K., Sundrum A. (2015). Detection of SARA in the transition period of dairy cows. J. Dairy Sci..

[94] Bossen D., Mertens D.R., Weisbjerg M.R. (2008). Influence of fermentation methods on neutral detergent fiber degradation parameters. J. Dairy Sci..

[95] Hall M.B. (2013). Dietary starch source and protein degradability in diets containing sucrose: Effects on ruminal measures and proposed mechanism for degradable protein effects. J. Dairy Sci..

[96] Argyle J.L., Baldwin R.L. (1989). Effects of amino acids and peptides on rumen microbial growth yields. J. Dairy Sci..

[97] Oba M., Allen M.S. (2003). Effects of corn grain conservation method on feeding behavior and productivity of lactating dairy cows at two dietary starch concentrations. J. Dairy Sci..

[98] Firkins J.L., Allen M.S., Oldick B.S., St-Pierre N.R. (1998). Modeling ruminal digestibility of carbohydrates and microbial protein flow to the duodenum. J. Dairy Sci..

[99] Erdman R.A., Varner M. (1995). Fixed yield responses to increased milking frequency. J. Dairy Sci..

[100] Stefanon B., Colitti M., Gabai G., Knight C.H., Wilde C.J. (2002). Mammary apoptosis and lactation persistency in dairy animals. J. Dairy Res..

[101] Murney R., Stelwagen K., Wheeler T.T., Margerison J.K., Singh K. (2015). The effects of milking frequency in early lactation on milk yield, mammary cell turnover, and secretory activity in grazing dairy cows. J. Dairy Sci..

[102] Bauman D.E., Mather I.H., Wall R.J., Lock A.L. (2006). Major advances associated with the biosynthesis of milk. J. Dairy Sci..

[103] Gardner N.H., Reynolds C.K., Phipps R.H., Jones A.K., Beever D.E. (2001). Effects of different diet supplements in the pre- and post-partum period on reproductive performance in the dairy cow. Fertility in the High Producing Dairy Cow.

[104] Van Knegsel A., van den Brand H., Dijkstra J., van Straalen W.M., Jorritsma R., Tamminga S., Kemp B. (2007). Effect of glucogenic *vs.* lipogenic diets on energy balance, blood metabolites, and reproduction in primiparous and multiparous dairy cows in early lactation. J. Dairy Sci..

[105] Hattan A.J. (2003). Energy Utilisation in High Yielding Dairy Cows.

[106] De Vries M.J., van der Beek S., Kaal-Lansbergen L., Ouweltjes W., Wilmink J.B. (1999). Modeling of energy balance in early lactation and the effect of energy deficits in early lactation on first detected estrus postpartum in dairy cows. J. Dairy Sci..

[107] Eastridge M.L. (2006). Major advances in applied dairy cattle nutrition. J. Dairy Sci..

[108] Van Arendonk J.A., Nieuwhof G.J., Vos H., Korver S. (1991). Genetic aspects of feed intake and efficiency in lactating dairy heifers. Livest. Prod. Sci..

[109] Beerda B., Ouweltjes W., Šebek L.B., Windig J.J., Veerkamp R.F. (2007). Effects of genotype by environment interactions on milk yield, energy balance, and protein balance. J. Dairy Sci..

[110] Rhoads M.L., Rhoads R.P., VanBaale M.J., Collier R.J., Sanders S.R., Weber W.J., Crooker B.A., Baumgard L.H. (2009). Effects of heat stress and plane of nutrition on lactating Holstein cows: I. Production, metabolism, and aspects of circulating somatotropin. J. Dairy Sci..

[111] Steffens A.B., de Boer S.F., Balm P.H. (1999). Impact of stress on animal intermediate metabolism. Stress in Physiology of Animals.

[112] Ingvartsen K.L. (2006). Feeding- and management-related diseases in the transition cow: Physiological adaptations around calving and strategies to reduce feeding-related diseases. Anim. Feed Sci. Technol..

[113] Veerkamp R.F., Brotherstone S. (1997). Genetic correlations between linear type traits, food intake, live weight and condition score in Holstein Friesian dairy cattle. Anim. Sci..

[114] Sutter F., Beever D.E. (2000). Energy and nitrogen metabolism in Holstein-Friesian cows during early lactation. Anim. Sci..

[115] Friggens N.C., Brun-Lafleur L., Faverdin P., Sauvant D., Martin O. (2013). Advances in predicting nutrient partitioning in the dairy cow: Recognizing the central role of genotype and its expression through time. Animal.

[116] Patton J., Kenny D.A., Mee J.F., O’Mara F.P., Wathes D.C., Cook M., Murphy J.J. (2006). Effect of milking frequency and diet on milk production, energy balance, and reproduction in dairy cows. J. Dairy Sci..

[117] Bradford B.J. Immunity and inflammation in transition cows. Proceedings of AABP Annual Meeting.

[118] Boutinaud M., Ben Chedly M.H., Delamaire E., Guinard-Flament J. (2008). Milking and feed restriction regulate transcripts of mammary epithelial cells purified from milk. J. Dairy Sci..

[119] Bell A.W., Burhans W.S., Overton T.R. (2000). Protein nutrition in late pregnancy, maternal protein reserves and lactation performance in dairy cows. Proc. Nutr. Soc..

[120] Lucy M.C. (2004). Mechanisms linking the somatotrophic axis with insulin: Lesson from the postpartum dairy cow. Proc. N. Z. Soc. Anim. Prod..

[121] Vernon R.G., Kasle M., Scholz H., Holtershinken M. (2002). Nutrient partitioning, lipid metabolism and relevant imbalances. Recent Developments and Perspectives in Bovine Medicine.

[122] Zhao F.-Q., Dixon W.T., Kennelly J.J. (1996). Localization and gene expression of glucose transporters in bovine mammary gland. Comp. Biochem. Physiol. B Biochem. Mol. Biol..

[123] Butler S.T., Marr A.L., Pelton S.H., Radcliff R.P., Lucy M.C., Butler W.R. (2003). Insulin restores GH responsiveness during lactation-induced negative energy balance in dairy cattle: Effects on expression of IGF-I and GH receptor 1A. J. Endocrinol..

[124] Lucy M.C. (2007). Fertility in high-producing dairy cows: Reasons for decline and corrective strategies for sustainable improvement. Soc. Reprod. Fertil. Suppl..

[125] Osborn O., Olefsky J.M. (2012). The cellular and signaling networks linking the immune system and metabolism in disease. Nat. Med..

[126] McNamara J.P. (1991). Regulation of adipose tissue metabolism in support of lactation. J. Dairy Sci..

[127] McNamara J.P., Harrison J.H., Kincaid R.L., Waltner S.S. (1995). Lipid metabolism in adipose tissue of cows fed high fat diets during lactation. J. Dairy Sci..

[128] Smith T.H., McNamara J.P. (1990). Regulation of bovine adipose tissue metabolism during lactation. 6. Cellularity and hormone-sensitive lipase activity as affected by genetic merit and energy intake. J. Dairy Sci..

[129] Lucy M.C., Jiang H., Kobayashi Y. (2001). Changes in the somatotrophic axis associated with the initiation of lactation. J. Dairy Sci..

[130] Rukkwamsuk T., Wensing T., Geelen M.J. (1998). Effect of overfeeding during the dry period on regulation of adipose tissue metabolism in dairy cows during the periparturient period. J. Dairy Sci..

[131] Rukkwamsuk T., Wensing T., Geelen M.J. (1999). Effect of overfeeding during the dry period on the rate of esterification in adipose tissue of dairy cows during the periparturient period. J. Dairy Sci..

[132] Locher L., Häussler S., Laubenthal L., Singh S.P., Winkler J., Kinoshita A., Kenéz Á., Rehage J., Huber K., Sauerwein H. (2015). Effect of increasing body condition on key regulators of fat metabolism in subcutaneous adipose tissue depot and circulation of non-lactating dairy cows. J. Dairy Sci..

[133] Katamoto H., Yukawa T., Shimada Y. (1996). Lipogenic and lipolytic activities in isolated adipocytes from cattle with fat necrosis. Res. Vet. Sci..

[134] Mann S., Yepes F.A.L., Overton T.R., Wakshlag J.J., Lock A.L., Ryan C.M., Nydam D.V. (2015). Dry period plane of energy: Effects on feed intake, energy balance, milk production, and composition in transition dairy cows. J. Dairy Sci..

[135] Andersen J.B., Ridder C., Larsen T. (2008). Priming the cow for mobilization in the periparturient period: Effects of supplementing the dry cow with saturated fat or linseed. J. Dairy Sci..

[136] Litherland N.B., da Silva D.N.L., Hansen W.P., Davis L., Emanuele S., Blalock H. (2013). Effects of prepartum controlled-energy wheat straw and grass hay diets supplemented with starch or sugar on periparturient dairy cow performance and lipid metabolism. J. Dairy Sci..

[137] Newman A., Mann S., Nydam D.V., Overton T.R., Behling-Kelly E. (2015). Impact of dietary plane of energy during the dry period on lipoprotein parameters in the transition period in dairy cattle. J. Anim. Physiol. Anim. Nutr. (Berl.).

[138] Overton T.R., Drackley J.K., Ottemann-Abbamonte C.J., Beaulieu A.D., Emmert L.S., Clark J.H. (1999). Substrate utilization for hepatic gluconeogenesis is altered by increased glucose demand in ruminants. J. Anim. Sci..

[139] Kreipe L., Vernay M.C.M.B., Oppliger A., Wellnitz O., Bruckmaier R.M., van Dorland H.A. (2011). Induced hypoglycemia for 48 hours indicates differential glucose and insulin effects on liver metabolism in dairy cows. J. Dairy Sci..

[140] Reynolds C.K., Aikman P.C., Lupoli B., Humphries D.J., Beever D.E. (2003). Splanchnic metabolism of dairy cows during the transition from late gestation through early lactation. J. Dairy Sci..

[141] Herdt T.H. (2000). Ruminant adaptation to negative energy balance. Influences on the etiology of ketosis and fatty liver. Vet. Clin. North Am. Food Anim. Pract..

[142] Hammon H.M., Stürmer G., Schneider F., Tuchscherer A., Blum H., Engelhard T., Genzel A., Staufenbiel R., Kanitz W. (2009). Performance and metabolic and endocrine changes with emphasis on glucose metabolism in high-yielding dairy cows with high and low fat content in liver after calving. J. Dairy Sci..

[143] Cadórniga-Valiño C., Grummer R.R., Armentano L.E., Donkin S.S., Bertics S.J. (1997). Effects of fatty acids and hormones on fatty acid metabolism and gluconeogenesis in bovine hepatocytes. J. Dairy Sci..

[144] Strang B.D., Bertics S.J., Grummer R.R., Armentano L.E. (1998). Effect of long-chain fatty acids on triglyceride accumulation, gluconeogenesis, and ureagenesis in bovine hepatocytes. J. Dairy Sci..

[145] Vazquez-Añon M., Bertics S., Luck M., Grummer R.R., Pinheiro J. (1994). Peripartum liver triglyceride and plasma metabolites in dairy cows. J. Dairy Sci..

[146] Cornish-Bowden A., Cárdenas M.L., Letelier J.-C., Soto-Andrade J., Abarzúa F.G. (2004). Understanding the parts in terms of the whole. Biol. Cell.

[147] Bannink A., France J., Lopez S., Gerrits W., Kebreab E., Tamminga S., Dijkstra J. (2008). Modelling the implications of feeding strategy on rumen fermentation and functioning of the rumen wall. Anim. Feed Sci. Technol..

[148] Sudakov K. (1997). The theory of functional systems: General postulates and principles of dynamic organization. Integr. Physiol. Behav. Sci..

[149] Oppenheim P., Putnam H., Feigl H. (1958). The unity of science as a working hypothesis. Concepts, Theories, and the Mind-Body Problem.

[150] Powell A., Dupré J. (2009). From molecules to systems: The importance of looking both ways. Stud. Hist. Philos. Biol. Biomed. Sci..

[151] Conway G.R. (1987). The properties of agroecosystems. Agric. Syst..

[152] Varela F.J. (1997). Patterns of life: Intertwining identity and cognition. Brain Cogn..

[153] Thompson P.B. (1997). The varieties of sustainability in livestock farming. EAAP Publ..

[154] Fox C.J., Hammerman P.S., Thompson C.B. (2005). Fuel feeds function: Energy metabolism and the T-cell response. Nat. Rev. Immunol..

[155] Wagner A. (2005). Robustness and Evolvability in Living Systems.

[156] Lucy M.C. (2003). Mechanisms linking nutrition and reproduction in postpartum cows. Reprod. Suppl..

[157] Buckley F., Dillon P., Rath M., Veerkamp R.F. (2000). The relationship between genetic merit for yield and live weight, condition score, and energy balance of spring calving Holstein Friesian dairy cows on grass based systems of milk production. J. Dairy Sci..

[158] Krause K.M., Oetzel G.R. (2006). Understanding and preventing subacute ruminal acidosis in dairy herds: A review. Anim. Feed Sci. Technol..

[159] Haerle C., Sundrum A. (2013). Animal health on the farm level 2. Communication: nutrient supply on bavarian dairy farms. Züchtungskunde.

[160] Holtenius K., Agenäs S., Delavaud C., Chilliard Y. (2003). Effects of feeding intensity during the dry period. 2. Metabolic and hormonal responses. J. Dairy Sci..

[161] Rastani R.R., Grummer R.R., Bertics S.J., Gümen A., Wiltbank M.C., Mashek D.G., Schwab M.C. (2005). Reducing dry period length to simplify feeding transition cows: Milk production, energy balance, and metabolic profiles. J. Dairy Sci..

[162] Gümen A., Rastani R.R., Grummer R.R., Wiltbank M.C. (2005). Reduced dry periods and varying prepartum diets alter postpartum ovulation and reproductive measures. J. Dairy Sci..

[163] Van Knegsel A., van der Drift S.G.A., Cermáková J., Kemp B. (2013). Effects of shortening the dry period of dairy cows on milk production, energy balance, health, and fertility: A systematic review. Vet. J..

[164] Chen J., Gross J.J., van Dorland H.A., Remmelink G.J., Bruckmaier R.M., Kemp B., van Knegsel A.T.M. (2015). Effects of dry period length and dietary energy source on metabolic status and hepatic gene expression of dairy cows in early lactation. J. Dairy Sci..

[165] McNamara S., Murphy J.J., O’Mara F.P., Rath M., Mee J.F. (2008). Effect of milking frequency in early lactation on energy metabolism, milk production and reproductive performance of dairy cows. Livest. Sci..

[166] Phyn C., Kay J.K., Rius A.G., Morgan S.R., Roach C.S., Grala T.M., Roche J.R. (2010). Review: Impact of short—Term alterations to milking frequency in early lactation. Proceedings of 4th Australian Dairy Science Symposium.

[167] Clark D.A., Phyn C., Tong M.J., Collis S.J., Dalley D.E. (2006). A systems comparison of once- *versus* twice-daily milking of pastured dairy cows. J. Dairy Sci..

[168] Stelwagen K., Phyn C.V.C., Davis S.R., Guinard-Flament J., Pomiès D., Roche J.R., Kay J.K. (2013). Invited review: Reduced milking frequency: Milk production and management implications. J. Dairy Sci..

[169] Carbonneau E., de Passillé A.M., Rushen J., Talbot B.G., Lacasse P. (2012). The effect of incomplete milking or nursing on milk production, blood metabolites, and immune functions of dairy cows. J. Dairy Sci..

[170] Soberon F., Ryan C.M., Nydam D.V., Galton D.M., Overton T.R. (2011). The effects of increased milking frequency during early lactation on milk yield and milk composition on commercial dairy farms. J. Dairy Sci..

[171] Oltenacu P.A., Algers B. (2005). Selection for increased production and the welfare of dairy cows: Are new breeding goals needed?. Ambio.

[172] Gordon F.J., Patterson D.C., Yan T., Porter M.G., Mayne C.S., Unsworth E.F. (1995). The influence of genetic index for milk production on the response to complete diet feeding and the utilization of energy and nitrogen. Anim. Sci..

[173] Tyrrell H.F., Brown A.C., Reynolds P.J., Haaland G.L., Bauman D.E., Peel C.J., Steinhour W.D. (1988). Effect of bovine somatotropin on metabolism of lactating dairy cows: Energy and nitrogen utilization as determined by respiration calorimetry. J. Nutr..

[174] Fourichon C., Seegers H., Beaudeau F., Verfaille L., Bareille N. (2001). Health-control costs in dairy farming systems in western France. Livest. Prod. Sci..

[175] Lawson L.G., Agger J.F., Lund M., Coelli T. (2004). Lameness, metabolic and digestive disorders, and technical efficiency in Danish dairy herds: A stochastic frontier production function approach. Livest. Prod. Sci..

[176] Lean I.J., van Saun R., DeGaris P.J. (2013). Energy and protein nutrition management of transition dairy cows: Metabolic diseases of dairy cattle. Vet. Clin. North Am. Food Anim. Pract..

[177] Von Keyserlingk M., Martin N.P., Kebreab E., Knowlton K.F., Grant R.J., Stephenson M., Sniffen C.J., Harner J.P., Wright A.D., Smith S.I. (2013). Invited review: Sustainability of the US dairy industry. J. Dairy Sci..

[178] Sundrum A., Konvalina P. (2012). “Healthy food” from healthy cows. Organic Farming and Food Production.

[179] Kahneman D. (2011). Thinking, Fast and Slow.

[180] Deutsche Forschungsgemeinschaft (2005). Future Perspectives of Agricultural Science and Research: Perspektiven der Agrarwissenschaftlichen Forschung.

[181] McNamara J.P. (2012). Ruminant Nutrition Symposium: A systems approach to integrating genetics, nutrition, and metabolic efficiency in dairy cattle. J. Anim. Sci..

[182] Friggens N.C., Newbold J.R. (2007). Towards a biological basis for predicting nutrient partitioning: The dairy cow as an example. Animal.

[183] Khan M.J., Hosseini A., Burrell S., Rocco S.M., McNamara J.P., Loor J.J. (2013). Change in subcutaneous adipose tissue metabolism and gene network expression during the transition period in dairy cows, including differences due to sire genetic merit. J. Dairy Sci..

[184] Ideker T., Galitski T., Hood L. (2001). A new approach to decoding life: Systems biology. Annu. Rev. Genomics Hum. Genet..

[185] Loor J.J., Bionaz M., Drackley J.K. (2013). Systems physiology in dairy cattle: Nutritional genomics and beyond. Annu. Rev. Anim. Biosci..

[186] Van Dien S., Schilling C.H. (2006). Bringing metabolomics data into the forefront of systems biology. Mol. Syst. Biol..

[187] Bannink A., France J., Dijkstra J., Te Pas M.F.W., Bannink A., Woelders H. (2011). Modeling approaches to link various levels of organization in animal physiology. Systems Biology and Livestock Science.

[188] Loor J.J., Bionaz M., Invermizzi G., Te Pas M.F.W., Bannink A., Woelders H. (2011). Systems biology and animal nutrition: Insights from the dairy cow during growth and the lactation cycle. Systems Biology and Livestock Science.

[189] Hampf M., Gossen M. (2007). Promoter crosstalk effects on gene expression. J. Mol. Biol..

[190] Kaern M., Elston T.C., Blake W.J., Collins J.J. (2005). Stochasticity in gene expression: From theories to phenotypes. Nat. Rev. Genet..

[191] Mossio M., Bich L., Moreno A. (2013). Emergence, closure and inter-level causation in biological systems. Erkenntnis.

